# Multi-omics analysis to decipher the molecular link between chronic exposure to pollution and human skin dysfunction

**DOI:** 10.1038/s41598-021-97572-1

**Published:** 2021-09-15

**Authors:** Namita Misra, Cécile Clavaud, Florent Guinot, Nasrine Bourokba, Stephanie Nouveau, Sakina Mezzache, Paul Palazzi, Brice M. R. Appenzeller, Arthur Tenenhaus, Marcus H. Y. Leung, Patrick K. H. Lee, Philippe Bastien, Luc Aguilar, Nükhet Cavusoglu

**Affiliations:** 1 Research and Innovation, L’Oréal SA, Aulnay Sous Bois, France; 2 Human Biomonitoring Research Unit, Luxembourg Institute of Health, Strassen, Luxemburg; 3 CentraleSupelec Laboratoire des Signaux et Systemes, Université Paris-Saclay, CNRS, Gif-sur-Yvette, France; 4 Brain and Spine Institute, Paris, France; 5 School of Energy and Environment, City University of Hong Kong, Kowloon, Hong Kong SAR, China

**Keywords:** Environmental impact, Computational biology and bioinformatics, Molecular biology, Biomarkers

## Abstract

Environmental pollution is composed of several factors, namely particulate matter (PM_2.5_, PM_10_), ozone and Ultra Violet (UV) rays among others and first and the most exposed tissue to these substances is the skin epidermis. It has been established that several skin disorders such as eczema, acne, lentigines and wrinkles are aggravated by exposure to atmospheric pollution. While pollutants can interact with skin surface, contamination of deep skin by ultrafine particles or Polycyclic aromatic hydrocarbons (PAH) might be explained by their presence in blood and hair cortex. Molecular mechanisms leading to skin dysfunction due to pollution exposure have been poorly explored in humans. In addition to various host skin components, cutaneous microbiome is another target of these environment aggressors and can actively contribute to visible clinical manifestation such as wrinkles and aging. The present study aimed to investigate the association between pollution exposure, skin microbiota, metabolites and skin clinical signs in women from two cities with different pollution levels. Untargeted metabolomics and targeted proteins were analyzed from D-Squame samples from healthy women (n = 67 per city), aged 25–45 years and living for at least 15 years in the Chinese cities of Baoding (used as a model of polluted area) and Dalian (control area with lower level of pollution). Additional samples by swabs were collected from the cheeks from the same population and microbiome was analysed using bacterial 16S rRNA as well as fungal ITS1 amplicon sequencing and metagenomics analysis. The level of exposure to pollution was assessed individually by the analysis of polycyclic aromatic hydrocarbons (PAH) and their metabolites in hair samples collected from each participant. All the participants of the study were assessed for the skin clinical parameters (acne, wrinkles, pigmented spots etc.). Women from the two cities (polluted and less polluted) showed distinct metabolic profiles and alterations in skin microbiome. Profiling data from 350 identified metabolites, 143 microbes and 39 PAH served to characterize biochemical events that correlate with pollution exposure. Finally, using multiblock data analysis methods, we obtained a potential molecular map consisting of multi-omics signatures that correlated with the presence of skin pigmentation dysfunction in individuals living in a polluted environment. Overall, these signatures point towards macromolecular alterations by pollution that could manifest as clinical sign of early skin pigmentation and/or other imperfections.

## Introduction

Pollution remains the world’s largest environmental threat to human health, responsible in 2017 for 15% of all deaths globally, and 275 million Disability-Adjusted Life Years, reported by *The Lancet Commission on Pollution and Health, 2017* and ambient air pollution kills more people around the globe than any other form of pollution (https://gahp.net/pollution-and-health-metrics/). Epidemiological and clinical studies have shown that short- and long-term exposure to particulate matter (PM_2.5_: Particle matter with aerodynamic diameter less than 2.5 microns, and PM_10_: Particle matter with aerodynamic diameter less than 10 microns) and ozone increase respiratory and cardiovascular morbidity and lead to the development of certain cancers^1–3^. Among all organs, skin is the most visible and there is accumulating scientific evidence that air pollution plays an important role in extrinsic aging^4,5^. Skin is a multi-layered tissue composed of a top layer, the stratum corneum, which is in direct contact with atmospheric pollutants, while the deeper layers, such as dermis, are exposed to diverse xenobiotics through systemic route. A major mechanism by which PM exert their detrimental effects is through the generation of oxidative stress^6,7^ which is an important contributor to extrinsic skin aging^8,9^. PM_2.5_ is often associated with toxic chemicals such as heavy metals or polycyclic aromatic hydrocarbons (PAHs), and some photo-reactive PAHs can induce strong oxidative stress under UVA exposure^10^. Clinical studies performed in China and elsewhere indicate towards premature skin aging phenotype such as pigmented spots and wrinkles^11,12^. In a previous study using in-vitro skin models and untargeted proteomics, we have shown that exposure to PAH (s) leads to disruption of several cellular processes^6^. However, the molecular mechanisms that link exposure and its impact on clinical manifestations remain to be deciphered.

The present work is part of a multi-parametric study, in which non-invasive facial skin samples (D-Squame) were collected from hundred and thirty-four healthy women living in two Chinese cities with different levels of exposure to pollution^13^. Exposure was determined by quantifying 39 parent PAHs and their mono-hydroxy metabolites in hair samples collected from the same individuals, according to a validated method providing information on the average internal dose of chemicals^13^. Each individual was also evaluated for a set of dermatological skin parameters. Microbiome profiling from all the subjects from both cities gave us the first insight into microbiome alteration resulting from pollution exposure^14^. To explore the mechanistic link between pollution exposure and its clinical manifestation, we considered several molecular characterizations. We first applied an untargeted metabolomic analysis on skin samples to characterize pollution-dependent biochemical events followed by a targeted proteomic analysis to complement the biochemical profiles generated through metabolomics. Finally, we complemented the multi-omics data by 16S and ITS amplicon sequencing. PAH quantification along with clinical evaluation of the same individuals completed this global characterization. These datasets were computed in a block structured multivariate analysis to propose a molecular map of human skin that links pollution exposure to skin dysfunction.

## Results and discussion

### Facial skin pigmentary disorders

To assess the effect of chronic exposure to pollution on exposed area of skin, we performed dermatological clinical assessment across 35 facial parameters on 67 women living in the city of Baoding (the most polluted area) and 67 women living in the city of Dalian (less polluted area), in China. The women had been living in respective cities for at least 15 years and were aged between 25 to 45 years. The clinical parameters analyzed on these women are presented in Suppl. Table 1. Briefly, each individual was evaluated for 10 major facial clinical themes/clusters that included 35 sub-conditions. Pigmentary disorders and wrinkles are the two clinical themes that were most significantly increased on facial skin of individuals from the polluted area. Shiny and dull skin were also modulated in a second instance (Suppl. Table 1). Previously, we have reported that individuals from the two cities differ significantly in PAH levels measured in hair shafts, suggesting different level of exposure to outdoor pollution^13^. These initial clinical evaluations were done on a larger population of 204 women and this difference remained significant for the study sub-population of 134 (Suppl_Fig. 1). Therefore, we calculated a PAH score (described in “Materials and methods” section) as a complementary factor to be analyzed together with the other datasets (clinical, metabolites, microbiome etc.) presented in this paper. As shown in Table 1, spread macules on forehead and cheeks and are significantly more prevalent (25.4% vs 6% and 56.7% vs 35.8% respectively) in women from the most polluted city of Baoding versus Dalian. A similar pattern is observed in the prevalence of spread macules on forehead and cheeks for women with PAH score higher than the median of the study population with regard to women with PAH score lower than the median (22.4% vs 9% and 53.7% vs 38.8% respectively). Previous studies have shown that the occurrence of some disorders of facial hyperpigmentation such as melasma, spots and lentigines are highest in individuals of skin type III-IV living in India and Southeast Asia^15^, these are also the geographical regions reporting heavy environmental PAH burden (https://gahp.net/pollution-and-health-metrics/). Results from our study show a similar trend. Spread macule (SP), a facial pigmentary condition is less known and has been described previously in South Asian population^16^ living in large metropolitan cities in India that are often the most polluted ones.

**Table 1:** Clinical facial signs for pigmentary disorders, their evaluation across Cities and PAH levels.

Clinical signs	Intensity	Dalian	Baoding	*p* value	PAH < MED	PAH > MED	*p* value
**Pigmentary disorders**							
Spread macules on forehead	No	94.03	74.63		91.04	77.61	
	Weak	2.99	17.91		7.46	13.43	
	Moderate	2.99	2.99	0.0029	1.49	4.48	0.0292
	Severe		4.48			4.48	
Spread macules on cheeks	No	64.18	43.28		61.19	46.27	
	Weak	32.84	32.84		34.33	31.34	
	Moderate	2.99	19.40	0.0024	2.99	19.40	0.0212
	Severe		4.48		1.49	2.99	
Hyperpigmented spots on forehead	No	26.87	20.90		25.37	22.39	
	Weak	73.13	76.12		74.63	74.63	
	Moderate		2.99	0.2844		2.99	0.4938
Simplex lentigo on cheek	No	25.37	13.43	0.0805	25.37	13.43	0.0805
	Yes	74.63	86.57		74.63	86.57	
Actinic lentigines on cheek	No	77.61	76.12	0.8377	82.09	71.64	0.1516
	Yes	22.39	23.88		17.91	28.36	
Melasma or melasma like on forehead	No	97.01	91.04	0.1447	97.01	91.04	0.1447
	Yes	2.99	8.96		2.99	8.96	
Melasma or melasma like on cheek	No	80.60	85.07	0.4919	80.60	85.07	0.4919
	Yes	19.40	14.93		19.40	14.93	

PAH median corresponds to the median of the PAH score, the first component of a principal components analysis (PCA) on log-normalized PAH measurements. *p* values were calculated using either unpaired two-samples Wilcoxon or chi^2^ tests depending on whether the clinical scores were ordinal or binary. The table was created using SAS version 9.4, SAS Institute Inc., Cary, NC, USA.

### Skin metabolite profile

**Figure 1:**
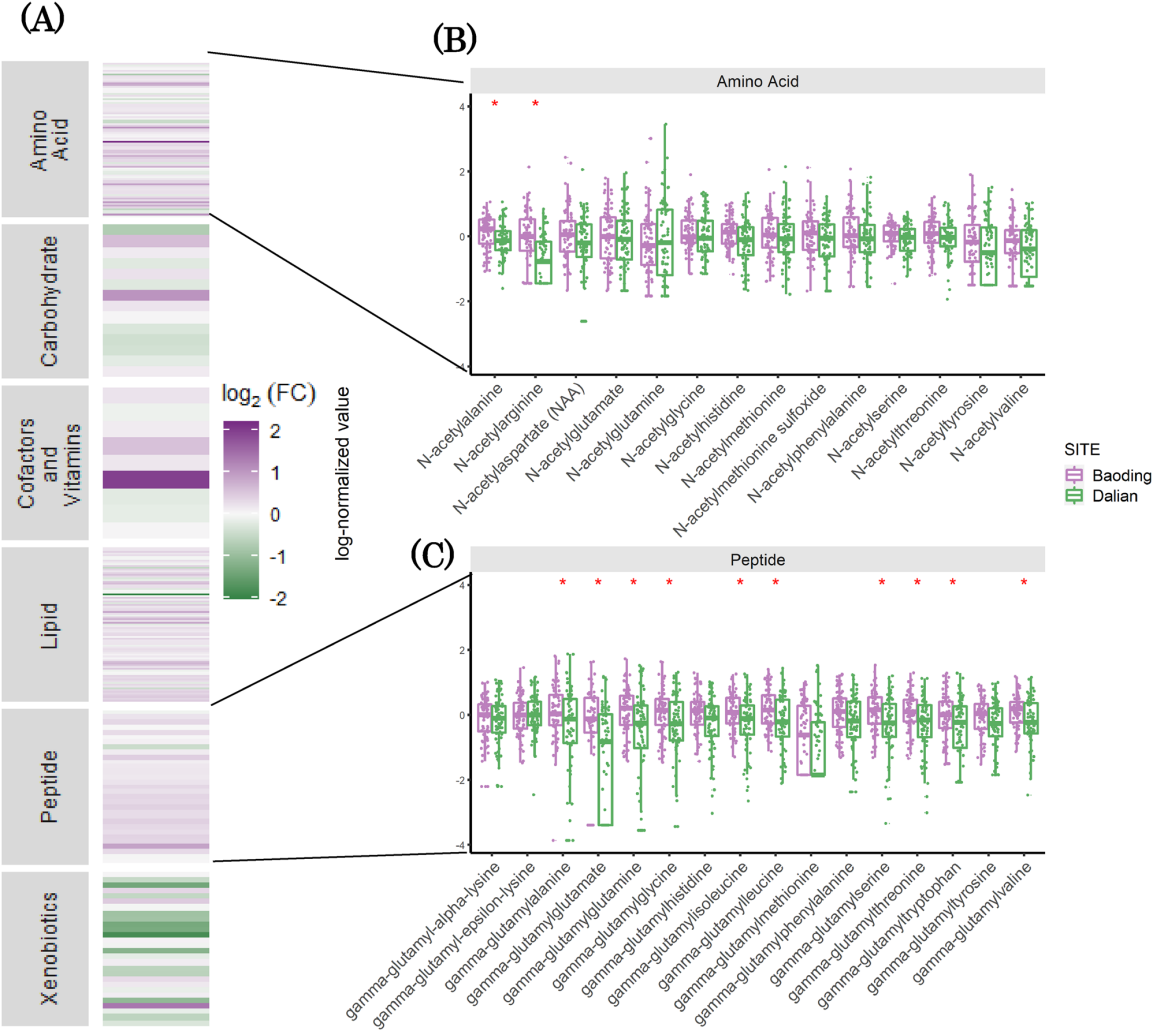
Metabolite pathways that are differentially modulated in skin samples of individuals from polluted city of Baoding. (**A**) Heatmap of log_2_ of Fold Change between Baoding (More Polluted) and Dalian (Less Polluted), positive values indicate a higher amount in Baoding and negative values a higher amount in Dalian. (**B**) Focus on N-acetyle amino acids with boxplots of log normalized values, colored by site. (**C**) Focus on gamma-glutamyl amino acids with boxplots of log normalized values, colored by site. A red star indicates, for a given metabolite, that the difference in mean between the 2 cities is significant (q-value < 0.05, t-test with correction for multiple testing using Benjamini–Hochberg method). Figures were created using the R software: R Core Team (2017), R Foundation for Statistical Computing, Vienna, Austria. URL https://www.R-project.org/.

**Figure 2:**
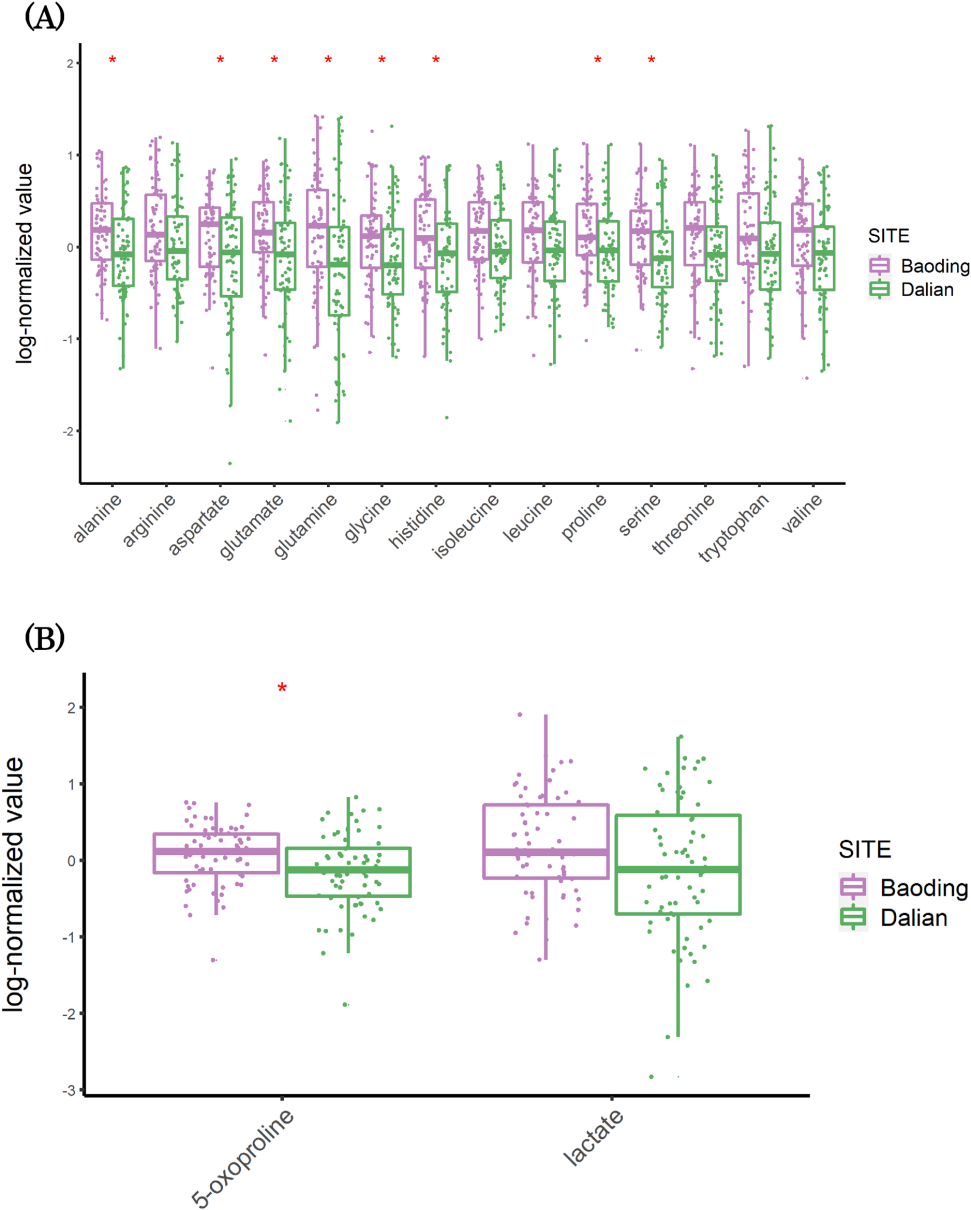
(**A**) Boxplots of log-transformed values of free amino acids (**B**) Boxplots of log-transformed values of NMF. A red star indicates, for a given metabolite, that the difference in mean between the 2 cities, Baoding (More Polluted) and Dalian (Less Polluted), is significant (q-value < 0.05, t-test with correction for multiple testing using Benjamini–Hochberg method). Figures were created using the R software: R Core Team (2017), R Foundation for Statistical Computing, Vienna, Austria. URL https://www.R-project.org/.

Untargeted metabolomic analysis on facial skin tape strips allowed the identification of a total of 350 metabolites. We first conducted a pathway enrichment analysis to explore if chronic exposure to pollution induced accumulation or perturbations in specific metabolic pathways. At a higher level, increased enrichment was observed in samples from Baoding that included amino acid and fatty acid metabolism (Fig. 1A and Suppl_Table 2). Xenobiotics of dietary origin appeared to be decreased compared to Dalian. Further analysis revealed elevated levels of N-acetyl amino-acids (Fig. 1B), gamma-glutamyl amino-acids (Fig. 1C), and urea cycle intermediates. *N*-acetyl amino acids are derived from proteins that have undergone post-translational acetylation or from free amino acids reacting with acetyl groups. Gamma-glutamyl transferase (GGT) is an enzyme that transfers the gamma-glutamyl moiety of glutathione to an acceptor that may be an amino acid or peptide^17^. The GGT system thus plays an important role in transporting amino acids and dipeptides into the cells as well as regulating the exchange of intra- and extracellular glutathione. Higher levels of gamma-glutamyl amino acids in the polluted group suggest an upregulated GGT system, perhaps to provide cells pyrrolidone carboxylic acid (PCA or 5-oxoproline), a natural moisturizing factor (NMF) released from GGCT enzyme following GGT activation^18^. NMF are the amino acids or their derivatives (PCA and urocanic acid, together with lactic acid, urea, citrate and sugars) that are produced due to filaggrin proteolysis and are found exclusively in stratum corneum (SC). Free amino acids (Fig. 2A), 5-oxoproline and lactic acid levels were elevated in Baoding versus Dalian (Fig. 2B) suggesting an upregulation of the filaggrin proteolysis pathway. To further explore the latter, we performed targeted protein analysis on proteins involved in skin barrier function. Surprisingly we observed decreased levels of CASP14, PADI1, TGM3 and GGCT. Latter, the gamma-glutamyl transferase enzyme is implicated in the last step of PCA synthesis, and since PCA levels were increased, this observation would suggest another mechanism that could be responsible for PCA increase. Interestingly, KLK7 a major enzyme in desquamation was decreased by 2 folds, suggesting a slow-down in surface renewal that could as a consequence lead to PCA and other amino acids accumulation. KLK5, another desquamation activating enzyme could be considered unchanged with a 1.2-fold increase. To further support this accumulation hypothesis, Filaggrin and Filaggrin2 which are the principal NMF providers, remained unaltered in skin samples from Baoding (Suppl_Fig. 2). Taken together, protein and metabolite analysis show a perturbation of molecular entities that contribute towards the skin barrier functioning and an enhanced repair/compensation in skin samples from Baoding. It appears that chronic exposure to pollution induces skin to be in a dynamic state to manage damage and repair simultaneously. Pollution induced effects on the skin barrier function have been reported by us previously using in-vitro models and untargeted proteomics^19^.

**Figure 3:**
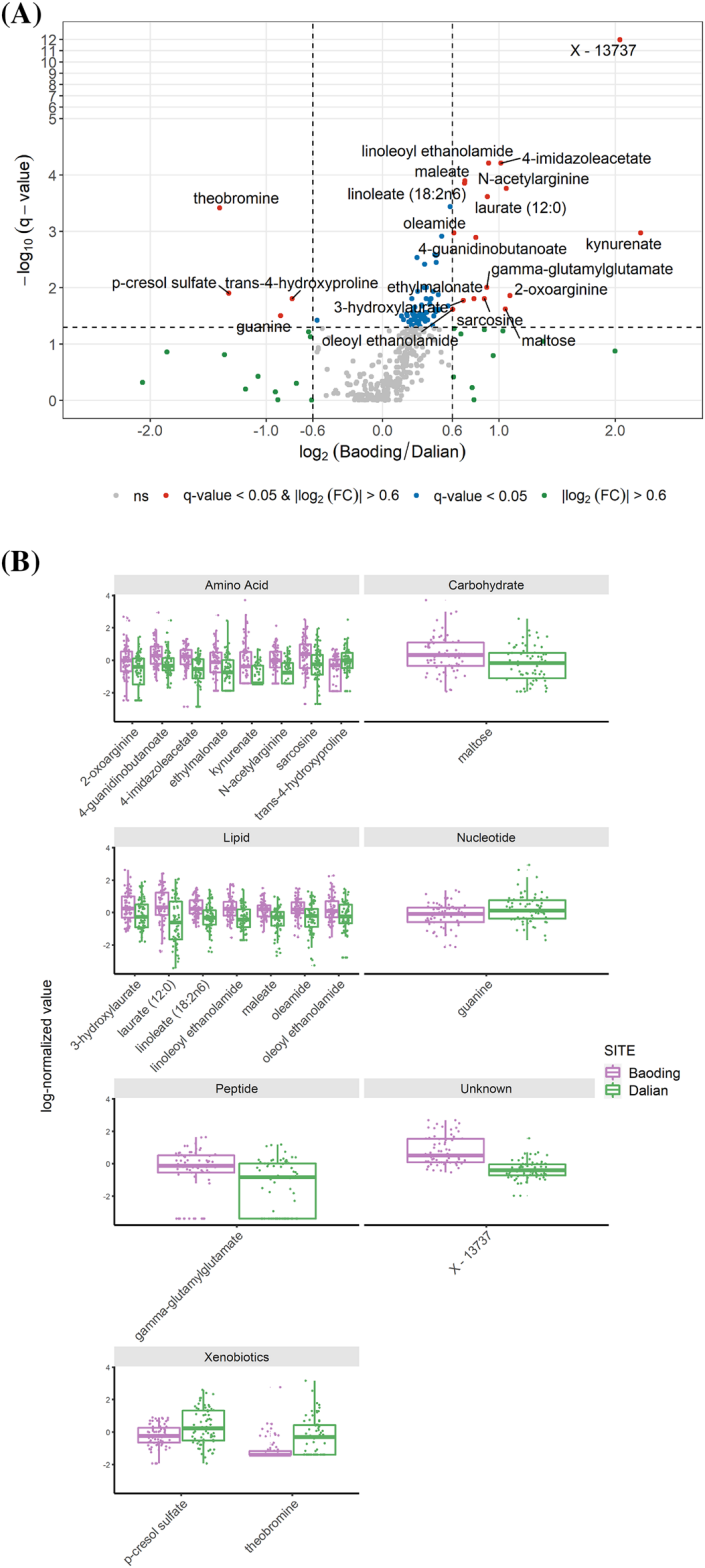
(**A**) Volcano plot representing the log2(FC) in x-axis and -log10(q − value) in y-axis. A threshold of 0.6 for the log2(FC) is added as a dotted vertical black line and a threshold of − log_10_(0.05) as a dotted horizontal black line for the q-value. For representation purpose, the y-axis scale has been squashed by a factor of 5 between 5 and 12. A metabolite is considered significantly modulated if its |log_2_(FC)|> 0.6 and q-value < 0.05 (in red and labelled on the figure). (**B**) Boxplots of significantly modulated metabolites, gathered by pathway, Baoding (More Polluted) and Dalian (Less Polluted). Figures were created using the R software: R Core Team (2017), R Foundation for Statistical Computing, Vienna, Austria. URL https://www.R-project.org/.

As a next step and in order to quantify the differences between the metabolomics profile between two cities more precisely and irrespective of classification into sub or super pathway, the log2-Fold change of all metabolites with Baoding as the reference population were calculated and represented as a cumulative distribution in Suppl_Fig. 3. Furthermore, to test the significance of the fold change, we performed a *t*-test for each of them with a volcano plot visualized in Fig. 3A.

To further investigate the relationship between the metabolite profiles and the city membership, we also conducted a multivariate analysis using a random forest classification model (see “Methods” section for more details). The variable importance values are shown in Suppl_Fig. 4A and further details on the RF model are given in Suppl_Fig. 4B,C.

When re-grouped by pathway (Fig. 3B), we find the most significant members of the different class of metabolites presented above and therefore allows for a deeper insight into the mechanisms that are most significantly associated with pollution exposure. Among lipids group, oleamide, oleoyl and linoleoyl ethanolamides are the fatty acid amides and are bioactive lipid signaling molecules. These are reported to be involved in a wide range of physiological responses in various tissues including skin conditions such as atopic and contact dermatitis^20^. Particulate matter leads to secretion of proinflammatory cytokines, e.g., tumor necrosis factor (TNF)-α, IL-1α, IL-8, and upregulation of matrix metalloproteinases 1, 2, and 9^21,22^, it is likely that bioactive fatty acids is the response against pollution induced inflammatory stress. Among other metabolites, 4-imidazole acetate, N-acetyl arginine and unknown X-13737 are significantly upregulated while p-cresol sulfate is downregulated (Fig. 3B). Detection of elevated levels of 4 imidazole-acetate indicates perturbation of Histidine metabolism and is discussed in the next section. Elevated levels of kynurenate indicates perturbation of Tryptophan metabolism and a focused analysis showed reduced indoxyl-3-sulfate in individuals in Baoding (Suppl_Fig. 5). The kynurenine pathway (KP) of tryptophan metabolism accounts for most of the tryptophan that is not involved in protein synthesis and includes compounds active in the nervous and immune systems. Kynurenine acts on the aryl hydrocarbon receptor (AhR), affecting metabolism of xenobiotics and affecting carcinogenesis. Limited studies have focused on the effects of acute ultraviolet exposure and the induction of the KP in human skin derived fibroblasts and keratinocytes. UV exposure is known to elicit an inflammatory component in skin cells, it is probable that the KP may be induced in these cells in response to UV exposure and this KP metabolites could be the mediators of inflammatory and anti-inflammatory responses^23^. We observed reduced indoxyl-3-sulfate in individuals living in the most polluted area. Several studies have identified tryptophan metabolite indole as a major extracellular metabolite produced by gut bacteria such as E. coli and interestingly, indoxy-3-sulfate has been characterized as AhR agonist^24^. P-cresol sulfate was found to be reduced in skin samples from Baoding (Fig. 2A). P-cresol has been previously reported as a biomarker for healthy aging^25^.

Another top marker in the volcano plot is the unknown metabolite “X-13737” (Fig. 3A,B). Upon further investigation, it was determined that the MS/MS spectrum for “X-13737” actually contained a mixture of ions from two metabolites, one being (S)-a-amino-omega-caprolactam and the other being a still unidentified molecule (Suppl_Fig. 6). Although the precise identity and contribution of this additional co-eluting unknown to the “X-13737” signal has not been fully verified, data suggest that it is one of the major actors that differentiates metabolomics profile of individuals living in one city from the other (Suppl_Fig. 4). The novelty of this finding is that caprolactam itself is a moderately toxic irritant, which has previously been regarded as a hazardous air pollutant. Although it is not known how (S)-a-amino-omega-caprolactam can be formed from caprolactam (or if it can be at all), the fact that caprolactam is a known air pollutant, it is noteworthy that the metabolomic data yielded an increase in signal for a spectrum which contains (S)-α-amino-omega-caprolactam in the skin of the women with higher exposure to pollutants as compared to the other ones.

### Skin metabolites and PAH exposure

**Figure 4:**
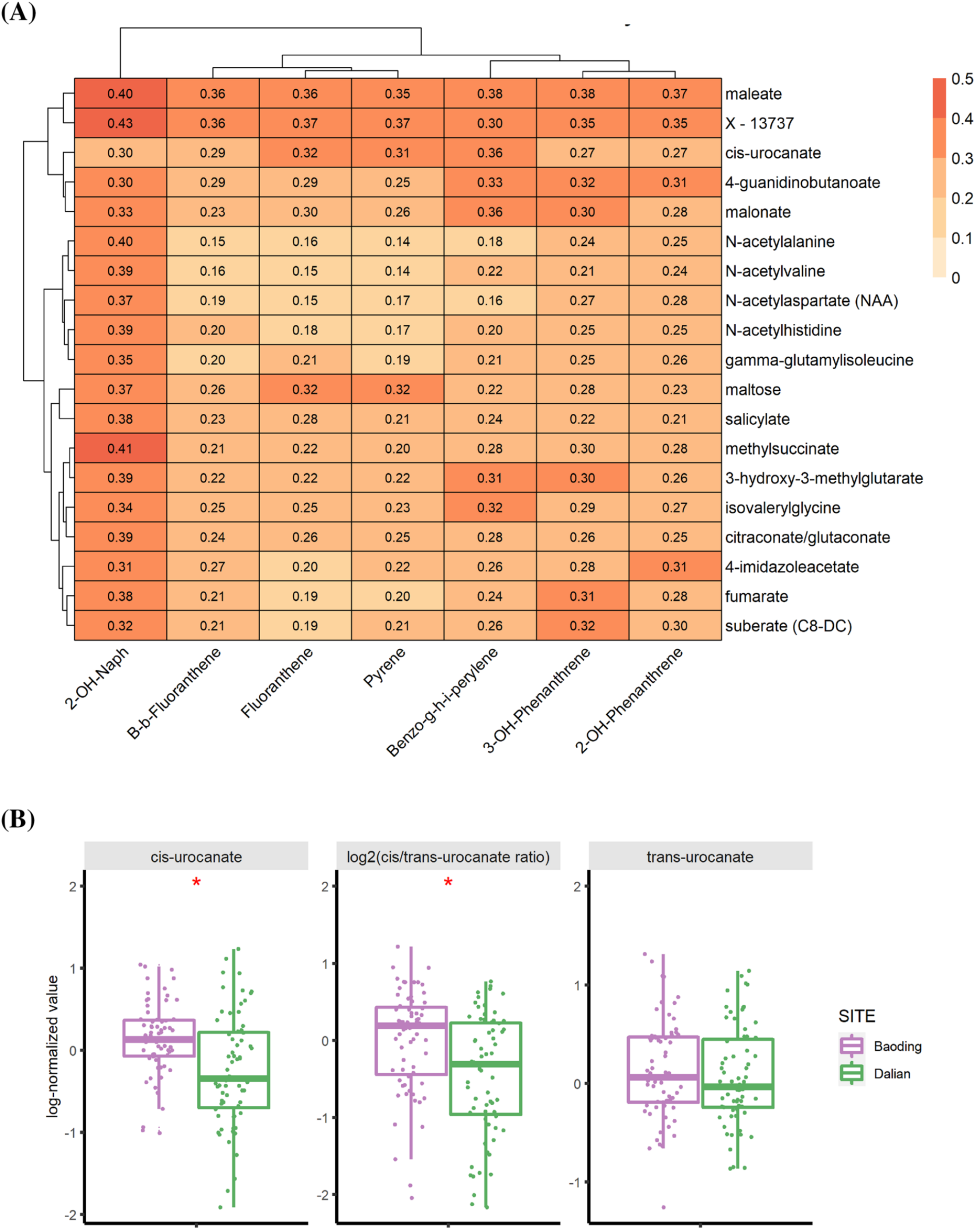
(**A**) Heatmap of cross-correlation between metabolites and PAHs selected by sparse Canonical Correlation Analysis (sCCA). The values in the heatmap correspond to Pearson’s correlation coefficient. (**B**) Focus on cis-and trans-urocanate metabolites with boxplots of the log-normalized values. We also represent the cis/trans urocanate ratio. A red star indicates that the difference in mean between the 2 cities, Baoding (More Polluted) and Dalian (Less Polluted), is significant (q-value < 0.05, t-test withcorrection for multiple testing using Benjamini–Hochberg method). Figures were created using the R software: R Core Team (2017), R Foundation for Statistical Computing, Vienna, Austria. URL https://www.R-project.org/.

From the analysis presented above, we saw significant differences in metabolomic profiles of skin from individuals living in polluted and non-polluted environments. To go one step further and to make a direct correlation between exposure and its molecular impact on skin, we carried out an analysis between PAHs concentrations, measured in individual hair samples, and skin SC metabolites using a sparse Canonical Correlation Analysis (sCCA) to identify sets of PAHs and metabolites that are correlated together. Hair analysis is increasingly used for the assessment of exposure, and several studies demonstrated that pollutant concentration in hair is representative of the body burden^26–28^. Hair analysis provides integrated information on chronic exposure covering up to several months (considering an average growth of one centimeter per month) and allows for the detection of both parent pollutants and their metabolites contrary to biological fluids^26^. Among the different correlations observed between PAH and skin metabolites, a positive correlation with N-acetyl amino acids was interesting (Fig. 4A). Acetylation is post-translational protein modifications that has multiple effects on cellular proteins and metabolites. Acetyl-coenzyme A donates acetyl group that can be post translationally attached to either the alpha amino group of N-terminus of proteins or to epsilon amino group of lysine residue. The reaction is catalyzed by N-acetyltransferase (NATs) and they are involved in a multitude of signaling pathways impacting diverse cellular functions^29^. The correlation between N-acetyl aa and PAH is intriguing. Previous studies have shown a link between exposure to particulate matter and histone acetylation^30^. Other studies show an association of NAT and cotinine levels from exposure to secondhand smoke^31^. Interestingly, we observed strong correlations of skin metabolites with 2OH Naphthalene, a PAH metabolite associated with smoking^32^. In conclusion, association between N-acetyl amino acids and PAH needs further investigation. Another pathway of interest was histidine metabolism since a correlation was observed between Cis-urocanate and 4 imidazole-acetate and PAH (Fig. 4A). Previous studies have shown that Benzo[a]pyrene perturbs histidine metabolism in human lung epithelial cells^33^. We observed elevated levels of Cis-urocanate in individuals from the polluted city (Fig. 4B). Trans-urocanate levels remained unchanged between the two cities resulting in elevated Cis/trans ratio in polluted city of Baoding (Fig. 4B). Histidine is deaminated by histidase to form trans-urocanate. While trans-urocanate acts as photo-protectant, cis-urocanate is implicated to suppress the immune response, leading to UV-induced immunosuppression.

### Impact of skin microbiome on metabolites and vice-versa

On the skin surface, there is a complex interplay between the host metabolism and its microbiota. It cannot be ruled out that a subset of metabolites presented above are either directly produced or are altered by the skin microflora. Indeed, skin microbiome plays an important role in maintaining cutaneous health and the skin microflora is constantly adapting in response to intrinsic and extrinsic factors. Environment and therefore pollution exposure has the potential to influence the skin microflora and bacterial isolated from the human skin have been shown to degrade PAHs and related xenobiotic compounds^33^.

In a previous study based on the same cohort, we have shown that an increase in Shannon diversity was correlated to PAH levels score. This observation was further supported by identification of pathways that point towards host-microbe interaction and degradation of aromatic compounds in a metagenomics analysis on a limited number of individuals (n = 32) who presented with very high and very low levels of PAH in their hair samples^14^. Here, to explore the influence of microbiome on skin metabolites and vice-versa, we used PLS prediction model and Pearson’s correlations between metabolites and microbiome on all the 134 individuals. In previous studies performed on gut, fecal metabolome has been reported to account for 68% of the variance in inter individual gut microbiome composition^34^, such comparisons are largely unexplored for skin. There has been a focus on persistence of the metabolites derived from skin care products^35^ or chemicals associated with urbanization^36^ or a higher level 3D modeling of the skin surface components^37^. In our study, for the first time, we have simultaneously analyzed endogenous metabolites and microbial diversity.

Using the prediction model, metabolome of the skin samples from the two cities accounted for 35% of the variance of bacterial diversity. Using the prediction model, metabolome of the skin samples accounted for only 14% of the variance of fungal skin diversity suggesting a higher contribution of the bacterial diversity to skin metabolites composition compared to fungi. Pearson’s correlations highlighted 42 metabolites significantly correlated to bacterial diversity, mainly amino acids (n = 19; including kynurenate), peptides (n = 10; including gamma-glutamylglutamate) and lipids (n = 11; including maleate and oleamide) (Suppl_Table 3). Two acetylated amino acids, N6-acetyllysine and N-delta-acetylornithine, were identified as the strongest correlated metabolites with a decrease in bacterial Shannon diversity. For fungal diversity, Pearson’s correlations were very weak (< 0.25), except for three metabolites: trans-4-hydroxyproline; linoleate (18:2n6) and nicotine (Suppl_Table 3). Our observations indicate a higher contribution of the bacterial diversity to metabolite composition compared to fungi.

**Figure 5:**
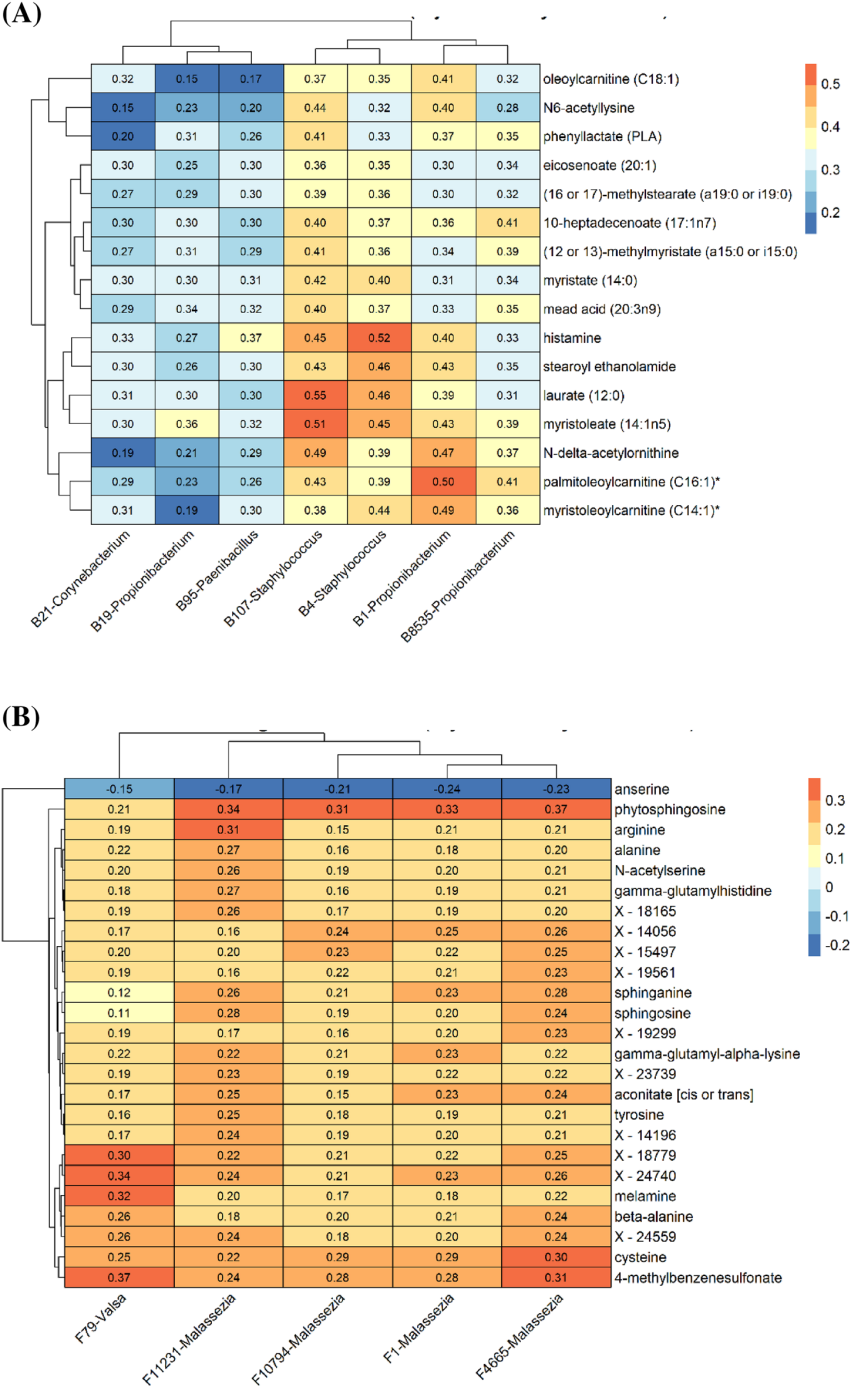
Heatmaps of the cross-correlation between OTU abundance and metabolites selected by sparse Canonical Correlation Analysis (sCCA), adjusted for city confounder. The values in the heatmap correspond to Pearson’s correlation coefficient. (**A**) bacterial OTU. (**B**) fungal OTU. Figures were created using the R software: R Core Team (2017), R Foundation for Statistical Computing, Vienna, Austria. URL https://www.R-project.org/.

To dig deeper into this analysis, we conducted sparse CCA (“Methods” section). As shown in Fig. 5A, mainly commensal bacterial taxa (*Propionibacterium* B1, B19 and B8535; *Staphylococcus* B107 and B4; *Corynebacterium* B21) were retrieved by sCCA with an exception of *Paenibacillus* B52, an environmental bacterium. A positive correlation was observed mainly with lipid metabolites (6/11 metabolites); laurate and myristoleate being the strongest correlated metabolites with *Staphylococcus* B107. Presence of free fatty acids could mirror triglyceride hydrolysis by microbial lipases as reported earlier^37,38^. A second group of metabolites was positively correlated with OTUs, gathering three acyl-carnitine (C18:1; C14:1; C16:1), that are not synthetized by bacteria but can be a source of carnitine, a known nutrient and osmoprotectant, for bacteria^39^, suggesting that these acylcarnitine/carnitine favor skin commensals on the skin. This results contrasts with the gut metabolome^40^, where acylcarnitines were enriched in the case of gut dysbiosis. Only two amino acids were positively correlated with *Propionibacterium* and *Staphylococcus* OTUs that corresponded to the same metabolites associated to bacterial diversity: N6-acetyllysine and the arginine metabolite N-delta-acetylornithine. Although skin bacteria such as *Staphylococcus* have the capacity to produce these amino acids, to our knowledge there are no existing data supporting their contribution to the skin metabolome content, nor link with skin dysbiosis. Furthermore, two metabolites were positively correlated with bacterial taxa: histamine and phenyllactate^30^. Histamine is a well-known mediator of the allergic reaction^41^. Histamine is not only synthesized in mast cells but is also produced by commensal microorganisms in the gut under physiological conditions^42,43^. We can postulate a similar case on the skin, since *Propionibacterium* and *Staphylococcus* have the capacity to secrete histamine too^44^. PLA is a biopreservative produced by propionic acid bacteria such as *Propionibacterium*, lactic acid bacteria (LAB)^45^, and *Paenibacillus.* Given the symbiotic association of *Paenibacillus* with a stingless bee, associated with antimicrobial activity of PLA, our observation raise the possibility that PLA produced by skin commensals protect the skin from external pathogens^46^. Skin metabolome analysis presented in previous sections showed tryptophan pathway to be modulated in individuals living in polluted city of Baoding (Suppl_Fig. 5). Specifically, we observed reduced indoxyl-3-sulfate, an aryl hydrocarbon receptor (AhR) agonist, in individuals living in more polluted city and is reported to be significantly lower in the skin of AD patients^47^. The present analysis did not show significant correlation between indoxyl-3-sulfate and bacterial taxa. Additional analysis at the functional level using metagenomics is underway to investigate the link between tryptophan metabolism and the microbiome.

The majority of fungal taxa selected with sCCA corresponded to *Malassezia* OTU (Fig. 5B). They have distinct correlations profiles compared to bacteria with either amino acids/peptides (arginine, alanine, gamma-glutamylhistidne), the long-chain sphingoid bases phytosphingosine, sphinganine and sphingosine and unknown metabolites. Phytosphingosine, sphingosine and sphinganine are main constituents of the stratum corneum ceramides. In addition to their role in barrier function, these molecules are reported to favor the growth of *Malassezia* sp. while inhibiting the growth of opportunistic pathogens such as *Candida albicans*^48^; which could explain the correlation observed in the present study.

Comparing skin microbiota and skin metabolome highlighted a strong link between sebum degradation and bacterial taxa (*Propionibacterium* and *Staphylococcus*), this also revealed a potential link between these taxa and the presence of carnitine, histamine and PLA on skin, which could represent new factors involved in the commensal—host homeostasis. As the correlation between bacterial diversity and metabolites was stronger, we decided to focus on bacterial taxa for further analysis.

### Multi-omics signature of pollution exposure induced clinical signs

The results presented so far allowed us to characterize individual parameters such as clinical, metabolites, microbiome and their correlation to pollution exposure. This analysis revealed certain mechanisms linked to barrier function as illustrated by metabolites and proteins and/or sebum degradation from microbiome analysis. To investigate the possible link between these diverse biological parameters, exposure and a visible perceptible clinical skin dysfunction, we conducted a multiblock data analysis (see “Methods” section for details) that concerns the analysis of several sets of variables (blocs) observed on same individual. The need to analyze conjointly the different data sets to discover their main sources of covariation requires the use of very specific computational methods like the one recently proposed by Tenenhaus et al.^49^ in the framework of generalized canonical correlation analysis. This approach makes it possible to obtain a unique representation of individuals and descriptors in a consensus space, an essential step in the search for specific profiles/clusters based on classification^49,50^.

**Figure 6:**
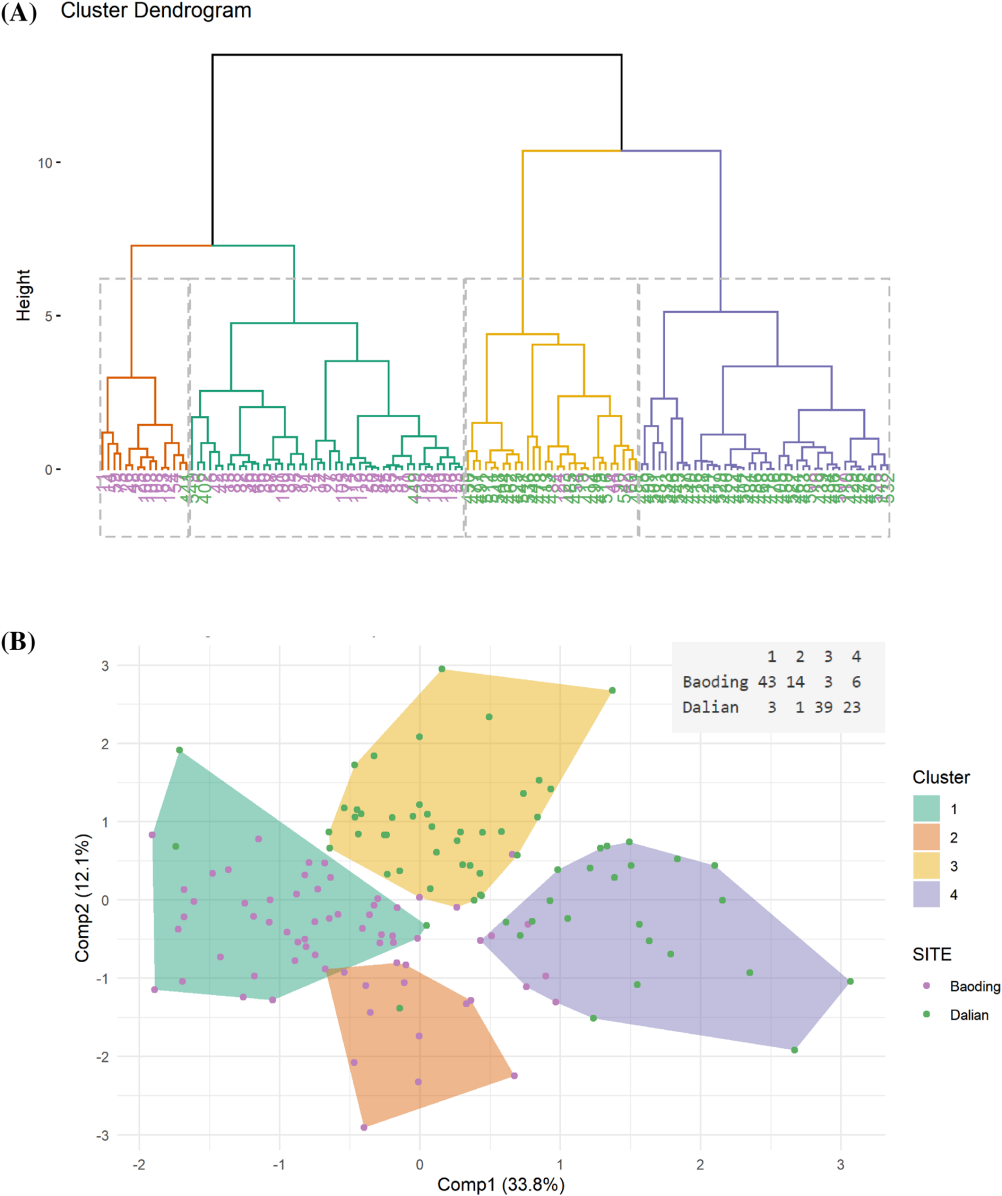
(**A**) Cluster dendrogram of the hierarchial clustering performed on the consensus space constructed by MAXVAR-A model from the PAH, metabolites and Bacteria OTUs selected by sCCA. Individuals are colored by cities. Based on the height of the gap between 2 consecutive level of the dendrogram, we chose to construct 4 clusters of individuals. (**B**) Representation of hierarchical clustering on the MAXVAR-A consensus space, with colored convex hull around cluster. The two first component of the consensus space represent 45.9% of the total variability. The table in the upper-right of the figure represents the repartition of individuals between the 2 cities, Baoding (More Polluted) and Dalian (Less Polluted), for each cluster. Figures were created using the R software: R Core Team (2017), R Foundation for Statistical Computing, Vienna, Austria. URL https://www.R-project.org/.

**Figure 7:**
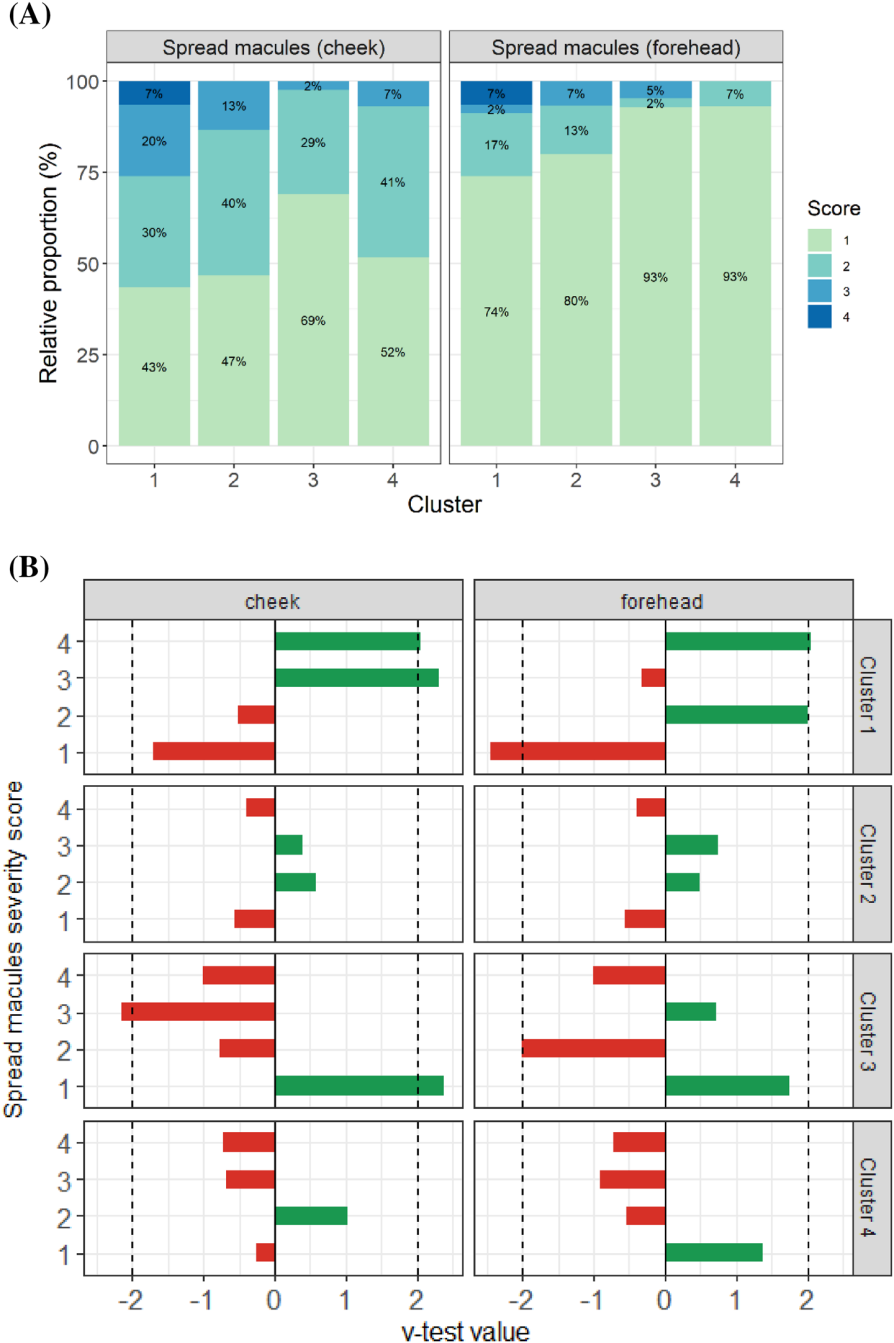
(**A**) Relative proportion of Spread Macules severity score in the consensus clusters. A score of 1 correspond to “no spread macules”, a score of 2 to “weak spread macules”, a score of 3 to “moderate spread macules” and a score of 4 to “severe spread macules”. (**B**) Characterization of consensus clusters by Spread Macules severity score using v-test statistic. In row we represent the consensus clusters and in column the location of the spread macule score on the face (cheek or forehead). A positive v-test value (in green) indicates that the severity score is in higher proportion in the cluster compare to the global population. A negative v-test value (in red) indicates that the severity score is in higher proportion in the cluster compare to the global population. The v-test value is considered significant if it reaches a threshold of 2 (black dotted vertical line on the plot). Figures were created using the R software: R Core Team (2017), R Foundation for Statistical Computing, Vienna, Austria. URL https://www.R-project.org/.

By following the methodology described in the method section, the entire population was partitioned into 4 distinct clusters as shown in Fig. 6. Cluster 1 and Cluster 3 are the largest and consist of 93.4% (n = 46) of Baoding and 92.8% (n = 42) of Dalian population respectively. Cluster 3 (n = 15) and Cluster 4 (n = 29) are smaller and have a mixed population. Each cluster has its unique molecular profile that consists of a list of metabolites, microbes and PAH and their derivatives. Each of these clusters was characterized by the variables differentially expressed in the clusters with regard to the whole study population, using a v-test (see “Methods” section for more details). We then obtained a subset of meaningful/relevant clinical parameter(s) for each of the clusters. It was interesting to note that among the 46 individuals of Cluster 1, 26 (57%) and 11 (25%) of them presented with spread macules on cheek and forehead respectively and that this cluster also had the highest scores for the severity for this pigmentary disorder (Fig. 7).

**Figure 8:**
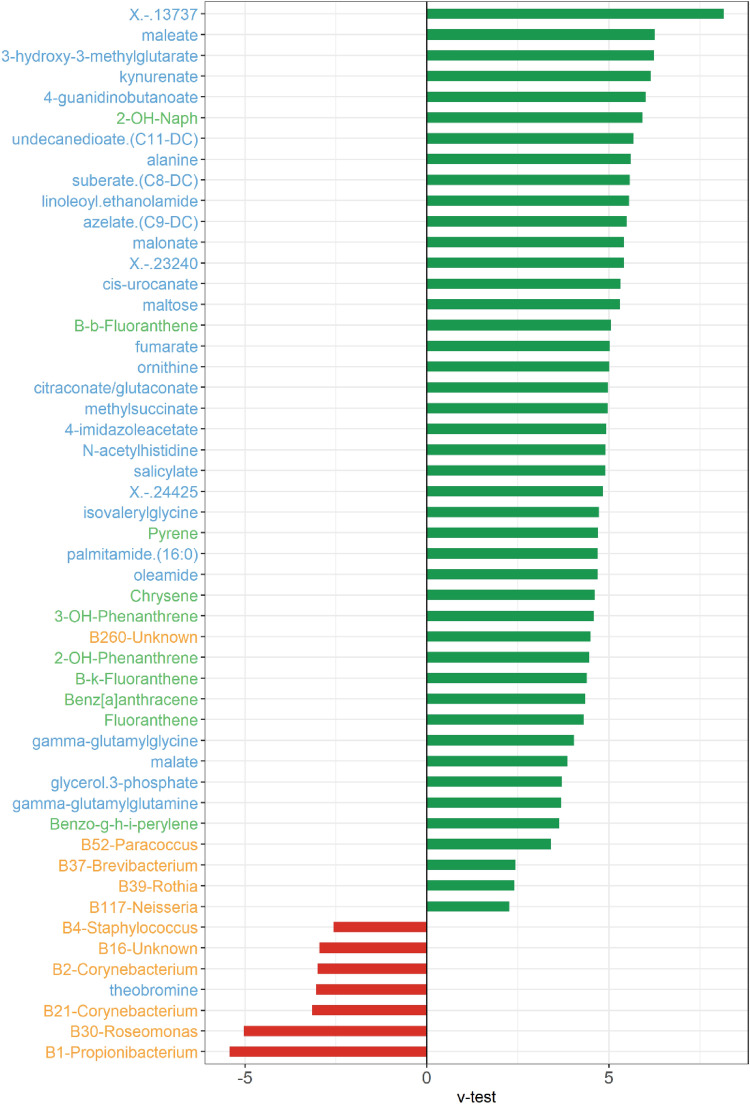
Focus on the characterization of the consensus cluster 1 by PAHs, metabolites and bacteria OTUs using v-test statistic. A positive v-test value (in green) indicates that the variable has mean value in the cluster higher than the global population. A negative v-test value (in red indicates that the variable has an average lower in the cluster higher than the global population. Only the variables with a significant v-test value are represented. The y-axis text is colored by type of variable (blue for metabolites, green for PAHs and yellow for bacterial OTUs). Figures were created using the R software: R Core Team (2017), R Foundation for Statistical Computing, Vienna, Austria. URL https://www.R-project.org/.

Cluster 2, 3 and 4, on the other hand, did not show spread macule as a dominant condition. In fact, these clusters showed mixed clinical characters (data not shown) and therefore, it was not possible to classify them into a precise clinical parameter category. Figure 8 represents the v-test values of the variables which are significantly modulated (under- or over-expressed) in the Cluster 1 and consists of 30 metabolites, 14 bacteria and 9 PAH and their derivative. We observed a good overlap of metabolites that were described earlier in this manuscript and were the most significantly modulated between polluted and less polluted cities. These include metabolites of tryptophan and histidine pathways (kynurenate, cis-urocanate and 4-imidazoleacetate), peptide (gamma-glutamyl glycine) and fatty acids (oleamide). Cluster 1 was characterized by a negative association with commensal bacteria taxa (Propionibacterium B1; Staphylococcus B4 and Corynebacterium B2 and B21) and positive associations were observed with two genera previously observed on aged skin (Neisseria B119; Rothia B39)^51^ and two genera linked to PAH (Brevibacterium B37^14^ and Paracoccus B52^52^). This analysis has allowed us to come up with a potential molecular ID or a road-map to investigate the association between chronic exposure to pollution and appearance of the pigmentary disorders. It is too early to speculate on the mechanisms and role of different entities (metabolites, Microbiome and PAH) that lead to this clinical condition. These are some of the questions that we are asking in our follow up studies through extensive metagenomics analysis and other clinical studies.

In conclusion, our study gives first insight into potential molecular perturbations and phenotype changes of skin due to chronic exposure to PAH, it also provides a roadmap of biological and clinical measurements and computational tools that could be applied to other similar studies including larger cohorts.

## Materials and methods

### Subject recruitment and sample collection

This multi-parametric clinical study was performed by the Sino-German Cosmetics Institute (Beijing) in agreement with the recommendations of the Declaration of Helsinki and was approved by the local ethics committee (Study n° 2015-033-DY-024, CAIQ Cosmetics Tech Center, Beijing, China, 19.08.2015). Informed written consent was obtained from all participants prior to any study-related procedure. Details of subject recruitment were described extensively elsewhere^13,14,28^. Briefly, study volunteers lived in two Chinese cities selected for being similar as regards UV exposure, geographical location and population lifestyle, but having different pollution levels, measured by monitoring stations as Air Quality Index (Suppl_Fig. 9). The selection was based on the data collected by the Chinese national air reporting system over several months, that showed different air pollution profiles of the two cities but that are located on comparable latitude and elevation and also have similar climate conditions^53^. Sixty-seven women were recruited in Dalian, the less polluted city, and 67 women in the more polluted city of Baoding. Age of subjects was limited to 25–45 years, to avoid major effects of hormonal variations during either puberty or menopause. Exclusion criteria included pregnancy, use of medications (e.g. antibiotics or antifungals), skin pathologies and current smoking. All participants provided information regarding health status, medical history, and daily habits. Each subject filled a self-administered questionnaire on skin care habits and time spent outdoors. Information on demographics and life-style of individuals is presented in Suppl_Table 4. Participants also underwent a clinical assessment by dermatologists with skin measurements, including scoring of skin signs and phenotypes. For microbiome analysis, cheek skin sampling was performed in a climate-controlled room at 22 °C and 60% humidity, as described previously^14^. Sterile cotton-tipped dry swabs were rubbed firmly on the cheek for 60 s to cover a surface area of 2 cm^2^. Cotton swabs were then placed into coded microfuge tubes, immediately flash frozen in liquid nitrogen, and stored at − 80 °C prior to further analyses. For metabolomics and proteomics D-Squame samples were collected from the cheek area as per manufacturer’s instructions. D-SQUAME is the registered name of a circular strip tape used to sample stratum corneum, Produced by CuDerm Corporation (Dallas, TX, U.S.A). For PAH analysis, a sample of hair was cut with stainless scissors from the occipital region of each subject, as described in^13,28^. Each sample comprised only the first 12 cm of hair starting from scalp, corresponding to around a year of hair growth before sampling. Hair samples were transferred into aluminium-foil paper and kept at room temperature until analysis.

### Metabolomic analysis of skin samples

The untargeted metabolomic analysis was performed at Metabolon, Inc (Morrisville, NC). Method of sample preparation and analysis described in this section was performed at Metabolon, Inc (Morrisville, NC) and has been described elsewhere before^54^. All samples were stored at − 80 °C until processed. Sample Preparation: Samples were prepared using the automated MicroLab STAR system from Hamilton Company. Several recovery standards were added prior to the first step in the extraction process for QC purposes. To remove protein, dissociate small molecules bound to protein or trapped in the precipitated protein matrix, and to recover chemically diverse metabolites, proteins were precipitated with methanol under vigorous shaking for 2 min (Glen Mills GenoGrinder 2000) followed by centrifugation. The resulting extract was divided into five fractions: two for analysis by two separate reverse phase (RP)/UPLC-MS/MS methods with positive ion mode electrospray ionization (ESI), one for analysis by RP/UPLC-MS/MS with negative ion mode ESI, one for analysis by HILIC/UPLC-MS/MS with negative ion mode ESI, and one sample was reserved for backup. All methods utilized a Waters ACQUITY (UPLC) and a Thermo Scientific Q-Exactive high resolution/accurate mass spectrometer interfaced with a heated electrospray ionization (HESI-II) source and Orbitrap mass analyser operated at 35,000 mass resolution. The MS analysis alternated between MS and data-dependent MS scans using dynamic exclusion. The scan range varied slightly between methods but covered 70–1000 m/z. Compounds were identified by comparison to library entries of purified standards or recurrent unknown entities based on retention time, molecular weight, preferred adducts and in-source fragments, as well as associated MS spectra and curated by visual inspection for quality control using software developed by Metabolon. Proteomic analysis by targeted MRM/SRM: Protein extraction, digestion, and mass spectrometry analyses were performed by the proteomics platform of the CHU de Quebec research center, Quebec, Qc, Canada. The samples (one D-Squame per subject) were resuspended in 550ul of extraction buffer (50 mM ammonium bicarbonate (ABC), 0.5% sodium deoxycholate, 50 mM DTT, protease inhibitor cocktail and 1uM pepstatin). After successive filtration and centrifugation, pellets were solubilized in 50µL of a 50 mM ABC/1% DOC solution for protein quantification by Bradford protein assay.

Tryptic digestion: Approximatively 10ug of each sample was heated at 95 °C for 5 min and proteins were reduced with 0.2 mM DTT at 37 °C 30 min and alkylated with 0.8 mM IAA (iodoacetamide) for 30 min at RT in the dark. The proteins were then digested with trypsin (1 ug) and incubated at 37 °C overnight.

SRM optmisation: Three peptides/protein of interest were selected by the three most intense transitions with an optimal collision energy. Selected peptides for SRM assay were synthesized with a heavy isotope on the C-ter amino acid ([13C6]-Lys and [13C6]-Arg) by ThermoFisher Scientific (Germany). Just before injection, 2.8 µL of sample are mixed with 2.8 µL of the stock heavy labeled synthetic peptide standard solution.

1 µg of each sample were analyzed on an Eksigent NanoLC 400 chromatography system (Sciex, Concord, Ontario) coupled online to a 6500QTRAPTM (ABSciex, Concord, Ontario) mass spectrometer with a nanospray ion source. The peptides were eluted with a linear gradient from 5 to 40% solvent B (A: 0.1% FA, B: ACN, 0.1% FA) in 30 min then 40 to 95% B in 10 min at 300 nL/min. Samples were injected randomly. Raw files were imported in Skyline v3.6 software for peak integration.

Results from Skyline were treated with excel, briefly the two more intense transitions for two peptides of each protein were used for quantification. The peak areas were normalized based on the heavey-labeled synthetic peptides. A normalisation factor was calculated and applied to the endogenous peptides. For each group, the mean of the sum of the normalized areas were calculated for each condition, then a ratio and a t-test were applied.

### Analysis of PAH and their metabolites in hair samples

Details on the methodology used for the analysis of hair PAH and metabolites have been previously reported^13^, here we describe briefly only the main steps. First, hair samples were washed and decontaminated, to remove any compounds on the hair surface without removing those incorporated in the bulk matrix via biological mechanisms and representative of the dose present in body. Then, hair samples were pulverized, hydrolyzed, extracted and analyzed according to well-established methods based on gas and liquid chromatography coupled with tandem mass spectrometry (GC–MS/MS and LC–MS/MS). PAH, PAH metabolites and nicotine/cotinine were analyzed separately, and quantified against table isotope labeled analogue. The method allowed for the analysis of 15 parent PAH (based on US-EPA priority list), 56 PAH monohydroxy-metabolites (all the commercially available standard), nicotine and cotinine. Limits of detection were also assessed.

### Analysis of skin microbiota

Detailed methodology for the analysis of skin microbiome has been reported earlier^14^. Briefly, gDNA was extracted from skin samples using the PowerSoil DNA isolation kit (MO BIO Laboratories, Carlsbad, CA, USA) following the manufacturer’s instruction with modifications as described previously, and each gDNA sample underwent triplicate PCR by primers targeting the bacterial 16S rRNA gene V1-3 region and the fungal ITS1 region. Following amplicon purification and indexing -PCR the library was prepared, and bacterial and fungal paired –end sequencing was performed on Illumina Miseq platform by SeqMatic LLC (Fremeont, CA, USA). 16S rRNA gene and ITS sequences underwent processing and bioinformatics analysis using USEARCH (v9.2.64) and QIIME (v1.9). Bacterial OTUs was obtained for the representative sequences after clustering at 97% sequence identity using the USARSE algorithm within USEARCH, against the SILVA database (128 release) and fungal OUT were interrogated against previously curated fungal database designed for skin microbiome analysis^55^. Following quality controls and after removing undesirable reads, a total of 9,656,916 and 14,649,172 bacterial and fungal reads were retained, respectively. Within-sample Shannon diversity (or alpha diversity) was estimated using the breakaway (v4.0) package in R v3.5.1 as reported earlier^14^. Correlations between metabolites and bacterial and fungal taxa were subsequently identified using a sCCA approach.

### Statistical methods

The article is structured into several parts, each with specific statistical approaches which are described below.

Association of clinical signs with city and PAH: Descriptive statistics for clinical signs have been tabulated by city and PAH groups. Clinical scores between cities or PHA groups were compared using a Wilcoxon test. The PAH score is the best univariate summary of all 39 PAHs measurements. It is the first principal component of a principal component analysis (PCA) based on the log-normalized PAH measurements.

The study being observational, *p* values must be interpreted more in an exploratory than confirmatory way, associations being potentially associated with confounding factors.

Metabolites and City: A pathway enrichment analysis has been performed. Metabolite pathways that are differentially modulated in skin samples of individuals from polluted versus less polluted cities are displayed using both heatmap of -log10 q-values (correction of *p* values using Benjamini–Hochberg multiple testing adjustment method) and boxplots by city of selected log normalized descriptors.

In order to evaluate the differences between the metabolomics profile between the two cities, irrespective of classification into sub or super pathway, a cumulative distribution of Log2-Fold change was calculated with the most polluted city, Baoding, as the reference population (Suppl_Fig. 3). The Fold changes have been calculated after removing the outliers having a value superior or equal to 3 times the interquartile range. Significance of these fold changes was tested using a t-test and the results are represented using a volcano plot which represents log2(FC) in x-axis and − log10(q-value) in y-axis. This bivariate representation allows to highlight the most relevant metabolites, i.e. those having a q-value < 0.05 and a |log2(FC)|> 0.6.

A complementary multivariate approach using a random Forest classification model was also used to confirm the robustness of the previous results (Suppl_Fig. 4A). The random forest allows to model the city membership as a non-linear multivariate function of the metabolites and to extract variable importance, i.e. the metabolites which contribute the most to the predictive accuracy of the model. We use the rfsrc function from the Random Forest SRC R package to perform the random forest classification model, more details on the model are given in Suppl_Fig. 4B. We also evaluate the predictive performance of the model on the Out-Of-Bag (OOB) samples, the accuracy results are given in Suppl_Fig. 4C. To compute the variable importance (VIMP) values we use a prediction error approach involving “noising-up” each variable in turn. VIMP for a variable X_j_ is the difference between prediction error when X_j_ is noised up by randomly permuting its values, compared to prediction error under the observed values^21^. Since VIMP is the difference between OOB prediction error before and after permutation, a large VIMP value indicates that misspecification detracts from the variable predictive accuracy in the forest. VIMP close to zero indicates the variable contributes nothing to predictive accuracy, and negative values indicate the predictive accuracy improves when the variable is mispecified. For the sake of clarity, we only represent the 20 metabolites with highest VIMP values in Suppl_Fig. 4A.

Metabolites and PAH: To make a direct comparison between exposure and its molecular impact on skin, a sparse Canonical Correlation Analysis (sCCA) has been carried out to identify sets of PAHs and metabolites that covary together. sCCA is a regularized version of canonical correlation analysis (CCA) used to study the relationship between two datasets while selecting only significant correlations^17^. If we have two datasets X and Y of random variables, and there are correlations among the variables, then the sCCA will find sparse linear combinations (components with null weights for non-relevant variable) of X and Y which have maximum correlation with each other. The sparsity parameters of sCCA were estimated using a permutation scheme (nperms = 500) with the MultiCCA.permute function from the PMA R package^17,]^^18^. The permutation procedure gave a *p* value of 0.004 associated with a correlation coefficient of 0.539 between the 1st sparse components of the two datasets. The 1st sparse components have 20 and 7 variables with non-null weights for metabolomics and PAH dataset respectively. The cross-correlation between the selected metabolites and PAHs was visualized using a heatmap representation.

Metabolites and skin microbiome: Correlation between the Shannon index of alpha bacterial or fungi diversity and the metabolites were estimated using Pearson correlation coefficient and ranked according to significance.

Prediction of Shannon index for bacterial or fungi diversity using the metabolites as independent variables was performed using a partial least squares regression with SIMCA software version 16.0, Umetrics, Umea, Sweden. sCCA was then performed to explore global associations between relative abundances of bacterial and fungal OTUs and metabolites (Fig. 5A,B). The sparsity parameters of sCCA were estimated using a permutation scheme (nperms = 500) with the MultiCCA.permute function from the PMA R package. The permutation procedure of the sCCA between the bacterial OTUs and the metabolites gave a *p* value < 0.001 associated with a correlation coefficient of 0.618 between the 1st sparse components of the two datasets. The 1st sparse components have 16 and 7 variables with non-null weights for the metabolomics and bacterial OTU datasets respectively. The permutation procedure of the sCCA between the fungal OTUs and the metabolites gave a *p* value of 0.206 associated with a correlation coefficient of 0.436 between the 1st sparse components of the two datasets. The 1st sparse components have 25 and 5 variables with non-null weights for the metabolomics and fungal OTU datasets respectively. The cross-correlation between the selected metabolites and OTUs was visualized using a heatmap representation.

Multiblock analysis: The need to analyze conjointly the different data sets to discover their main sources of covariation requires to generalize the sCCA approach to more than two groups. In that context, we use a sparse version of Regularized Generalized Canonical Correlation Analysis [ref] called Sparse Generalized Canonical Correlation Analyis (SGCCA) [ref]. S/RGCCA is a general component-based framework for a component-based framework for the integrative exploration of multimodal and high-dimensional data sets. S/RGCCA subsumes as special cases many multiblock component methods as special cases including MAXVAR-A (see Tenenhaus et al.^49^ for details). MAXVAR-A allows visualizing in a single space (the so-called consensus space), the relationships between variables belonging to the different blocks. We thus combine the metabolomics, PAH and bacterial OTU datasets using MAXVAR-A. Based on the 2 first components of the consensus space, we performed a hierarchical clustering to segment the population into 4 clusters (see Fig. 6A,B). The choice of 4 clusters was based on the visual evaluation of the dendrogram in Fig. 6A, more specifically by looking at the height of the gap between 2 consecutive level of the hierarchy. Each cluster was then characterized by clinical signs, metabolites, PAHs and bacterial OTUs using a v-test^20^, a procedure available from the catdes function of the Factominer R package. The v-test values allow determining if a variable is significantly over-represented or under-represented in the subgroup compared to the total population. For a continuous variable we test if the mean in a particular subgroup is different for the mean of the total population. For discrete/qualitative variables, we test if the proportion of a modality in a particular subgroup is over-expressed or under-expressed in the subgroup compared to the whole population. The results of v-test are shown in Fig. 7B for the spread macules clinical sign and in Fig. 8 for metabolites, PAHs and bacterial OTUs which are significantly modulated in cluster 1.

## Supplementary Information

Supplementary Figures.

Supplementary Table 1.

Supplementary Table 2.

Supplementary Table 3.

Supplementary Table 4.
