# Supplementary material for: Multi-omics analysis to decipher the molecular link between chronic exposure to pollution and human skin dysfunction
Source: Sci Rep. 2021 Sep 15;11:18302. doi: 10.1038/s41598-021-97572-1 (PMC8443591; doi:10.1038/s41598-021-97572-1)
Supplement: Supplementary file 1 — Supplementary Figures. [file 41598_2021_97572_MOESM1_ESM.docx]

**Supplementary Figures and Tables**

**Supplementary Figure 1**: A radar chart that shows a visual overall comparison of PAH descriptors between the two cities (Baoding, more polluted in purple and Dalian, less polluted in green). It consists of a sequence of equi-angular spokes, with each spoke representing one of the descriptors. The data length of a spoke has been chosen to be associated with the median value by city of the corresponding descriptor, with data log-transformed in order to make their distribution more symmetric and standardized to make them comparable on the chart. All statistical analyses in this part have been performed using SAS version 9.4, SAS Institute Inc., Cary, NC, USA


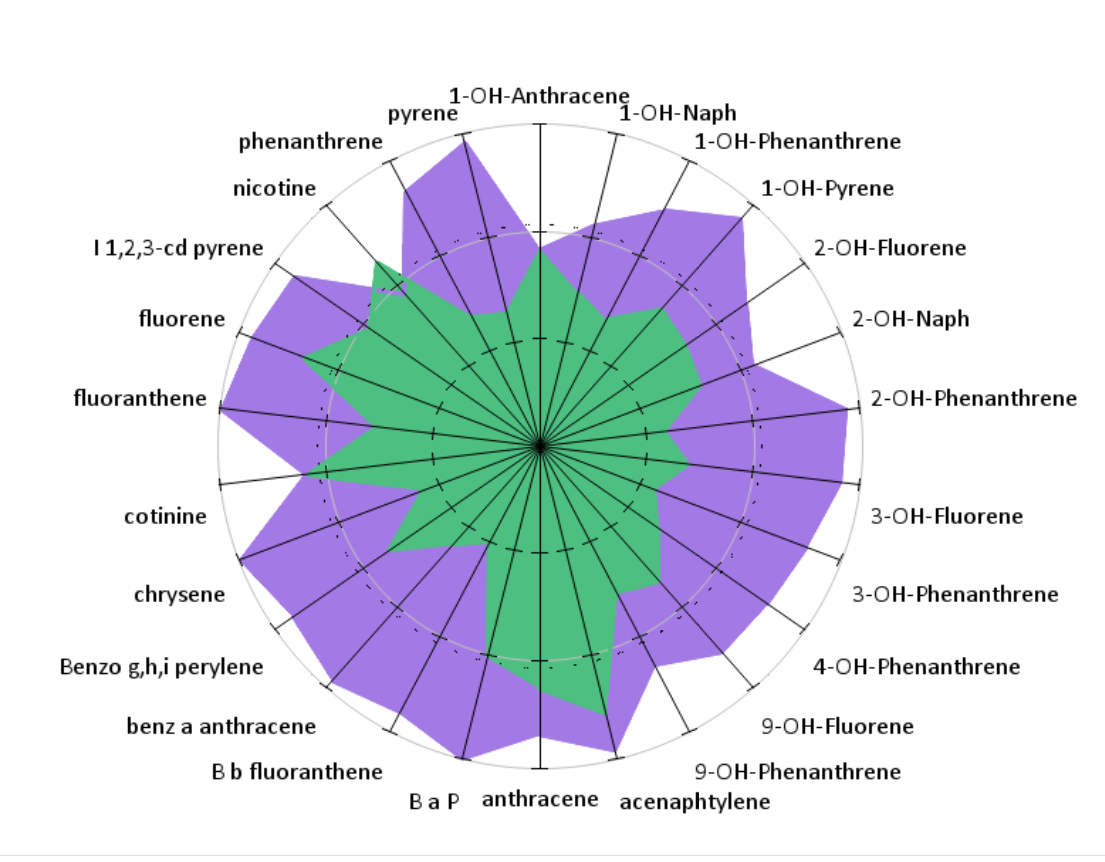


**Supplementary Figure 2**: Boxplots of log normalized values of some proteins associated with skin barrier function, colored by site. A red star indicates, for a given protein, that the difference in mean between the 2 cities, Baoding (More Polluted) and Dalian (Less Polluted), is significant (q-value < 0.05, t-test with correction for multiple testing using Benjamini-Hochberg method).


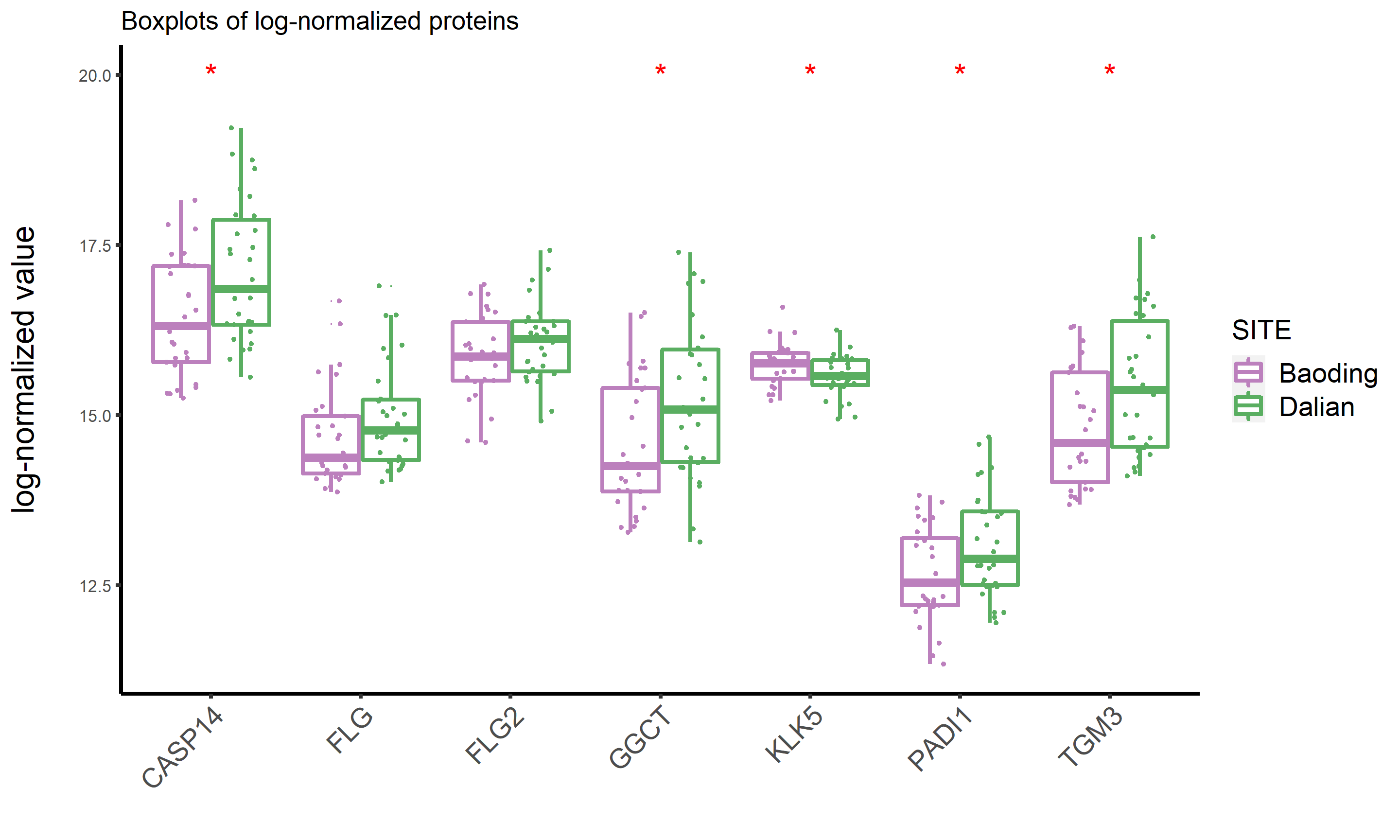


**Supplementary Figure 3**: Cumulative distribution of the log_2_ (Fold change) of all 350 metabolites with Baoding as the reference population. A value of 1 for the log_2_(FC) indicates that the biochemical is twice more abundant in the reference population (Baoding, more polluted). Metabolites having a |log2(FC)| > 0.6, i.e those being 1.5 times more abundant in one city or another are highlighted in colors (purple if FC > 0.6 and green if FC < -0.6). A star indicates that the fold change is significant (q-value < 0.05, t-test with correction for multiple testing using Benjamini-Hochberg method). The Fold changes have been calculated after removing the outliers having a value superior or equal to 3 times the interquartile range.


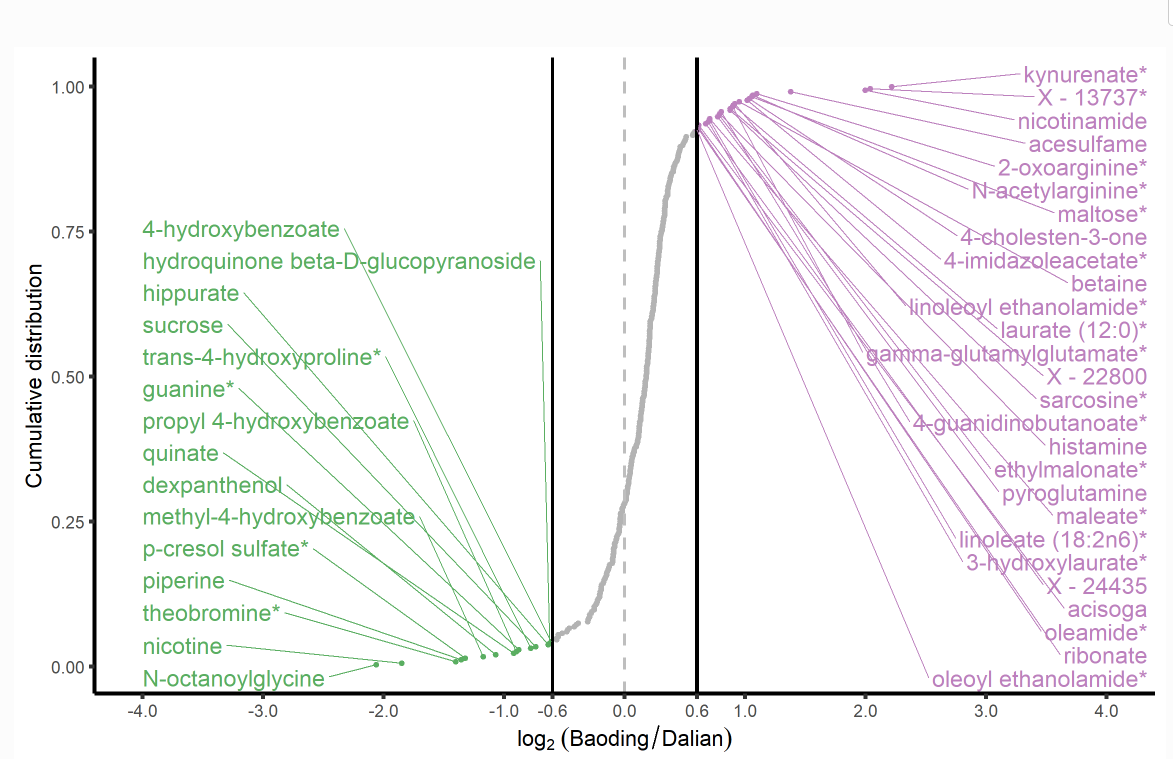


**Supplementary Figure 4**: **(A)** Variable importance (VIMP) plot from random forest classification model showing the top 20 metabolites with the highest VIMP values (VIMP values have been rescaled between 0 and 100). The metabolites names are colored by pathways. **(B)** Details on the random forest classification model. **(C)** Confusion matrix showing the classification error rate of the RF model on the Out-Of-Bag samples.


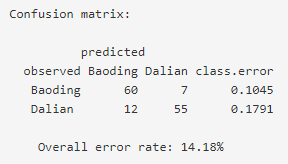

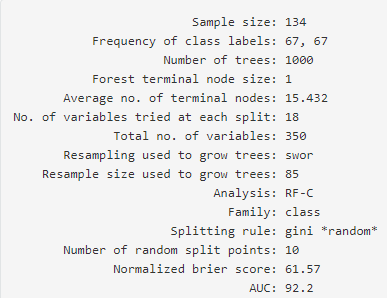

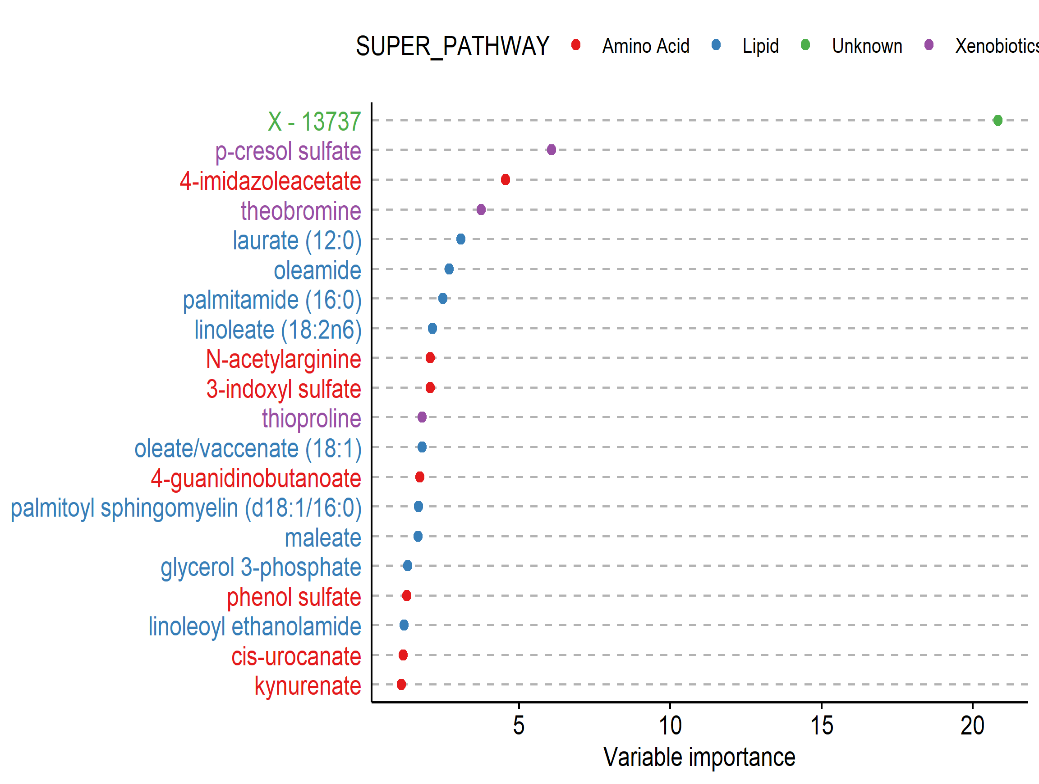


**(C)**

**(B)**

**(A)**

**Supplementary Figure 5**: Boxplots of log normalized values of metabolites belonging to tryptophan pathway, colored by site. A red star indicates, for a given protein, that the difference in mean between the 2 cities, Baoding (More Polluted) and Dalian (Less Polluted), is significant (q-value < 0.05, t-test with correction for multiple testing using Benjamini-Hochberg method).


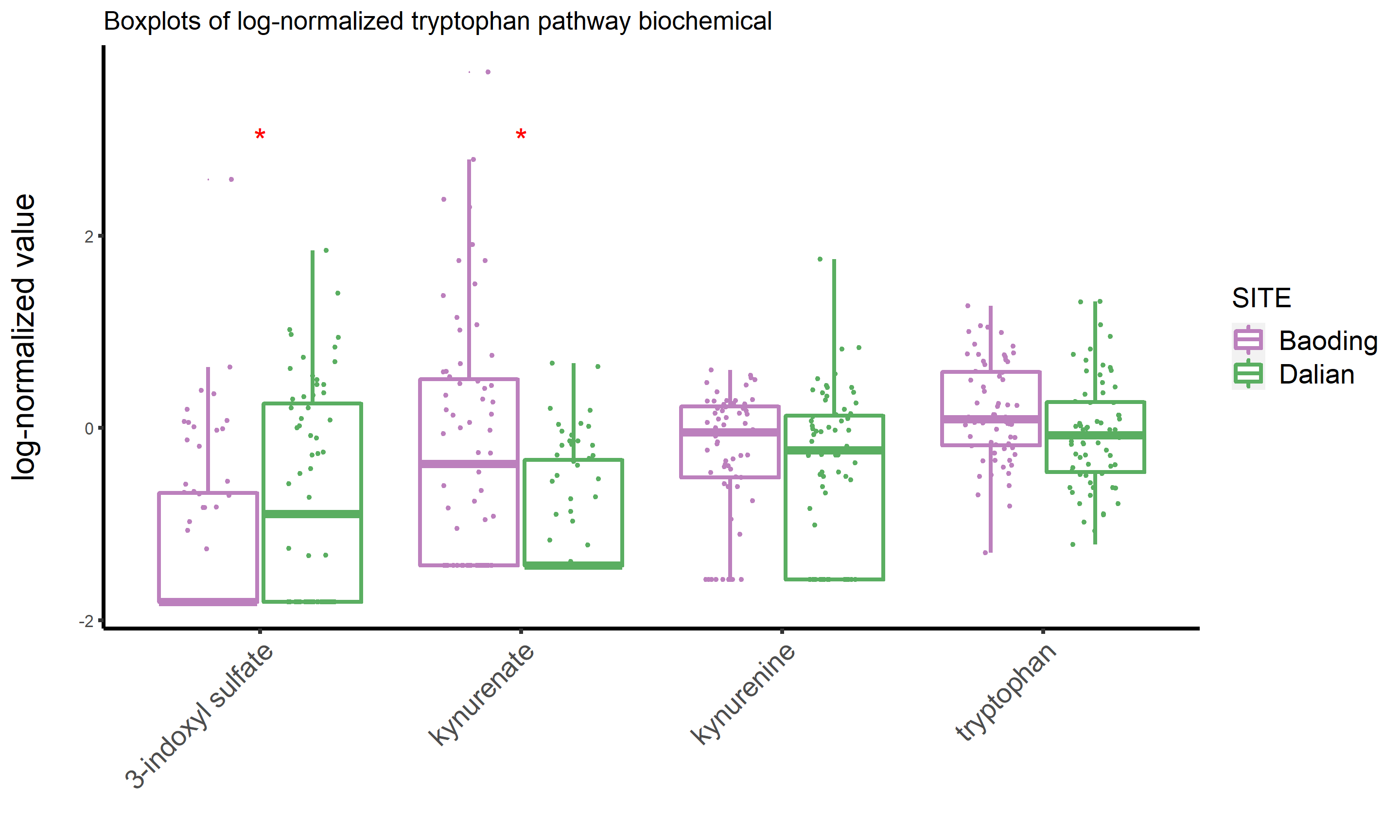


**Supplementary Figure 6**: Characterization of unknown X-13737. Chromatogram of pooled D-Squame samples and their corresponding spectra. Peak marked with blue arrow indicates S-amino-omega caprolactam, retention time =1,04mn and main fragment ddm2 mode = 84,09068 Da. Peak marked with red arrow marks Unknown compound at Rt =2,78mn. Main fragment ddm2 mode = 86,09625 Da. Yellow lines indicate the fragments obtained with the two peaks.


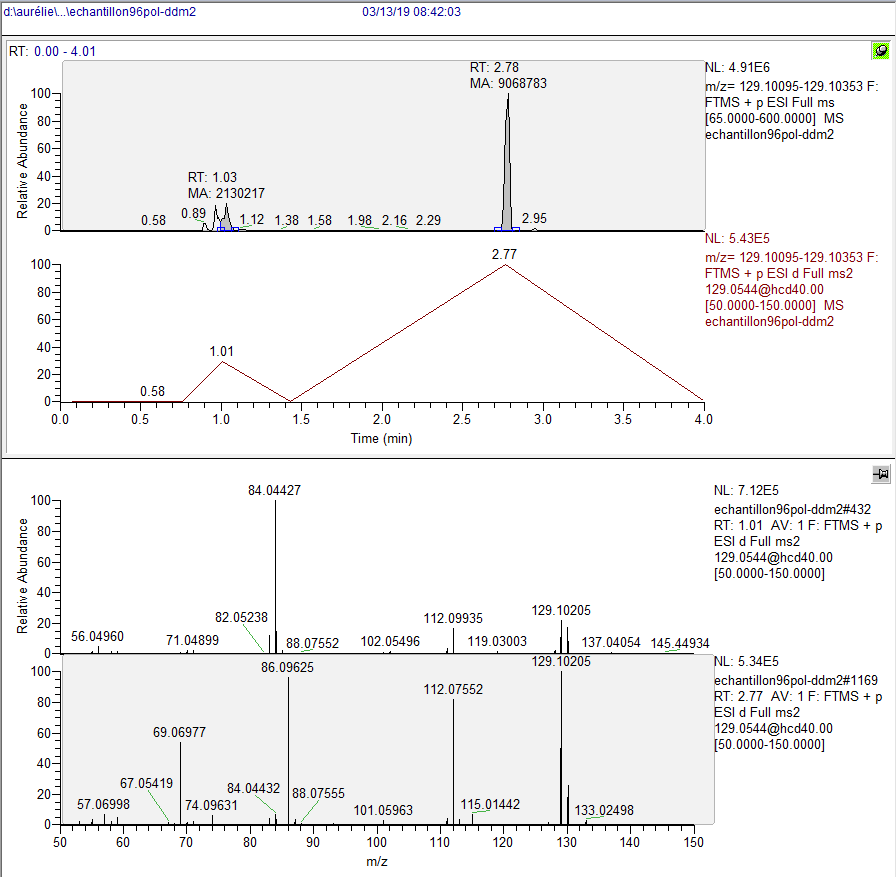

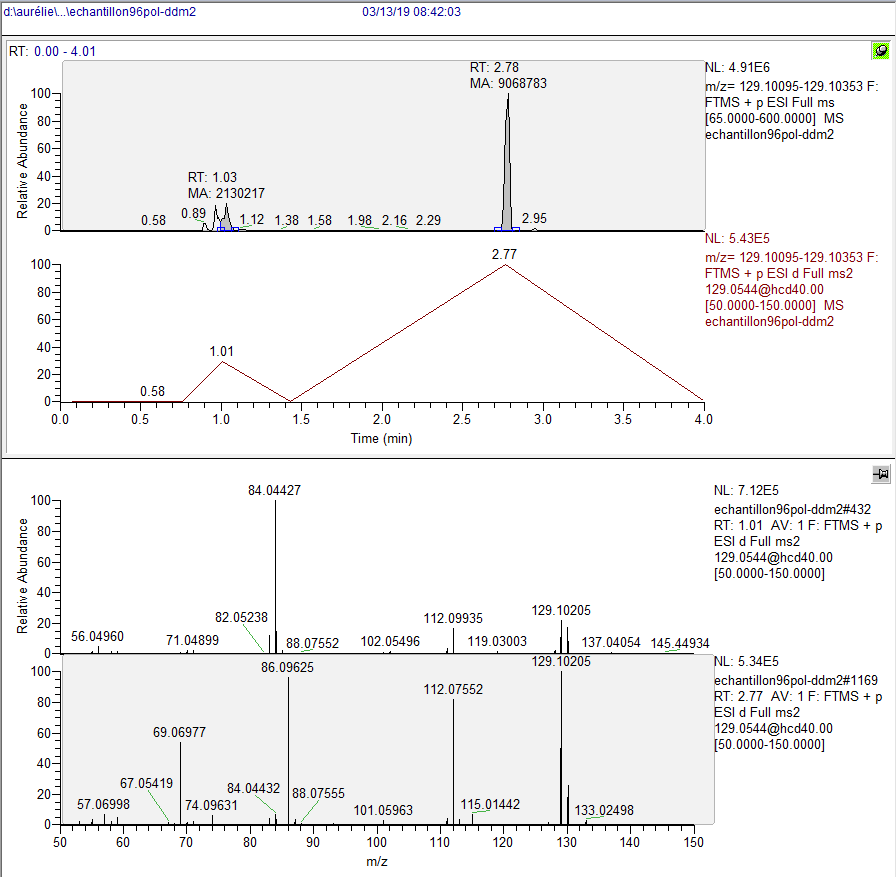


**Supplementary Figure 7**: Heatmap of cross-correlation between metabolites and bacterial OTUs abundance selected by sparse Canonical Correlation Analysis (sCCA) in Dalian (**A**) and Baoding (**B**). The values in the heatmap correspond to Pearson’s correlation coefficient.

**(A)**

**
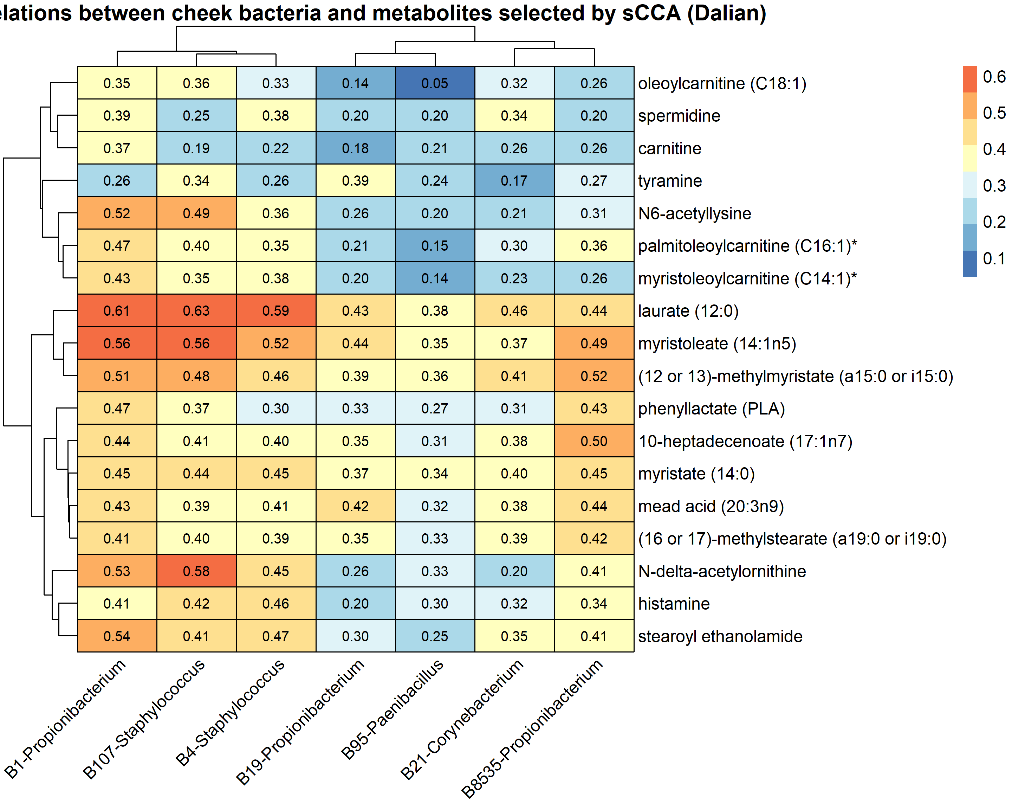
**

**(B)**

**
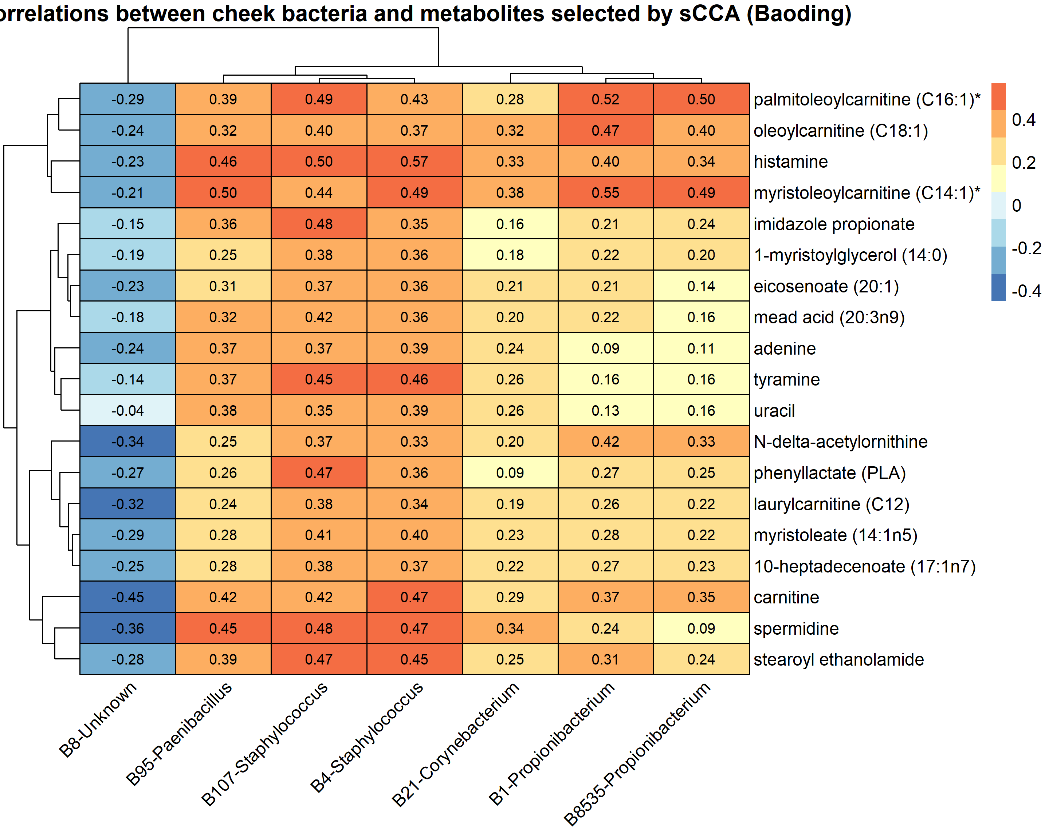
**

**Supplementary Figure 8**: Heatmap of cross-correlation between metabolites and fungal OTUs abundance selected by sparse Canonical Correlation Analysis (sCCA) in Dalian (**A**) and Baoding (**B**). The values in the heatmap correspond to Pearson’s correlation coefficient.

**(A)**

**
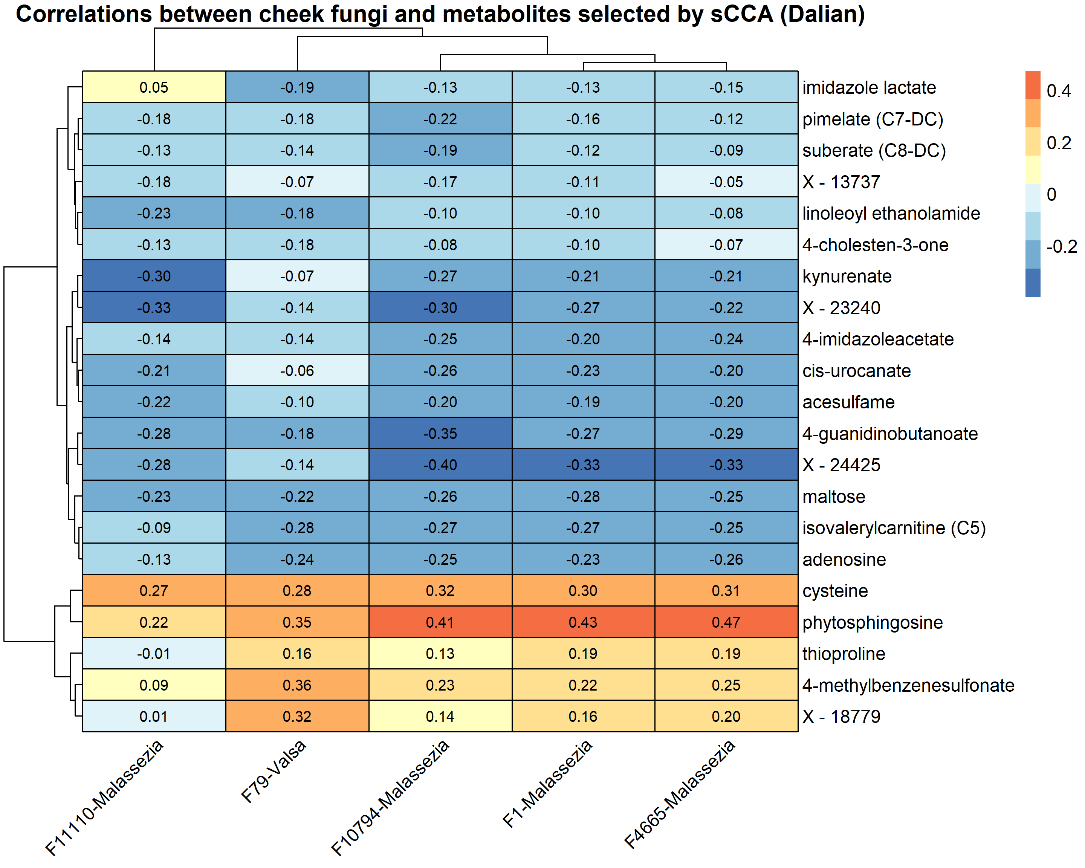
**

**(B)**


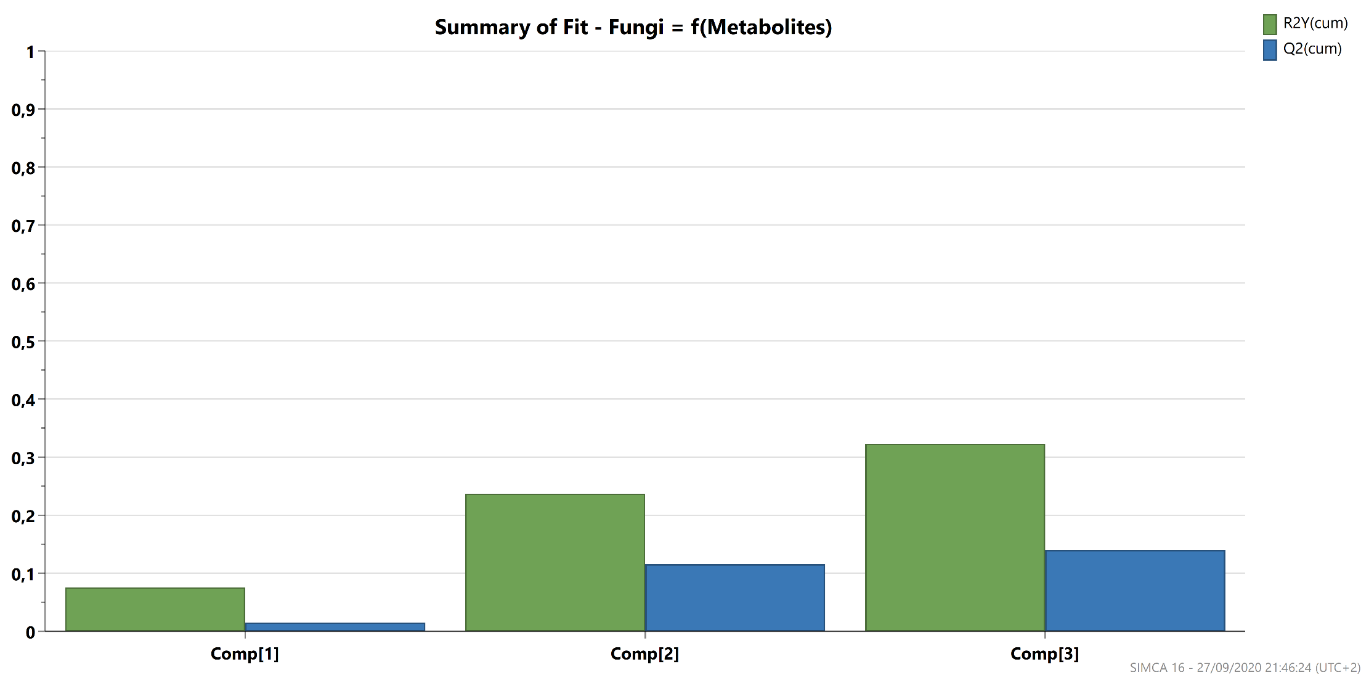
**
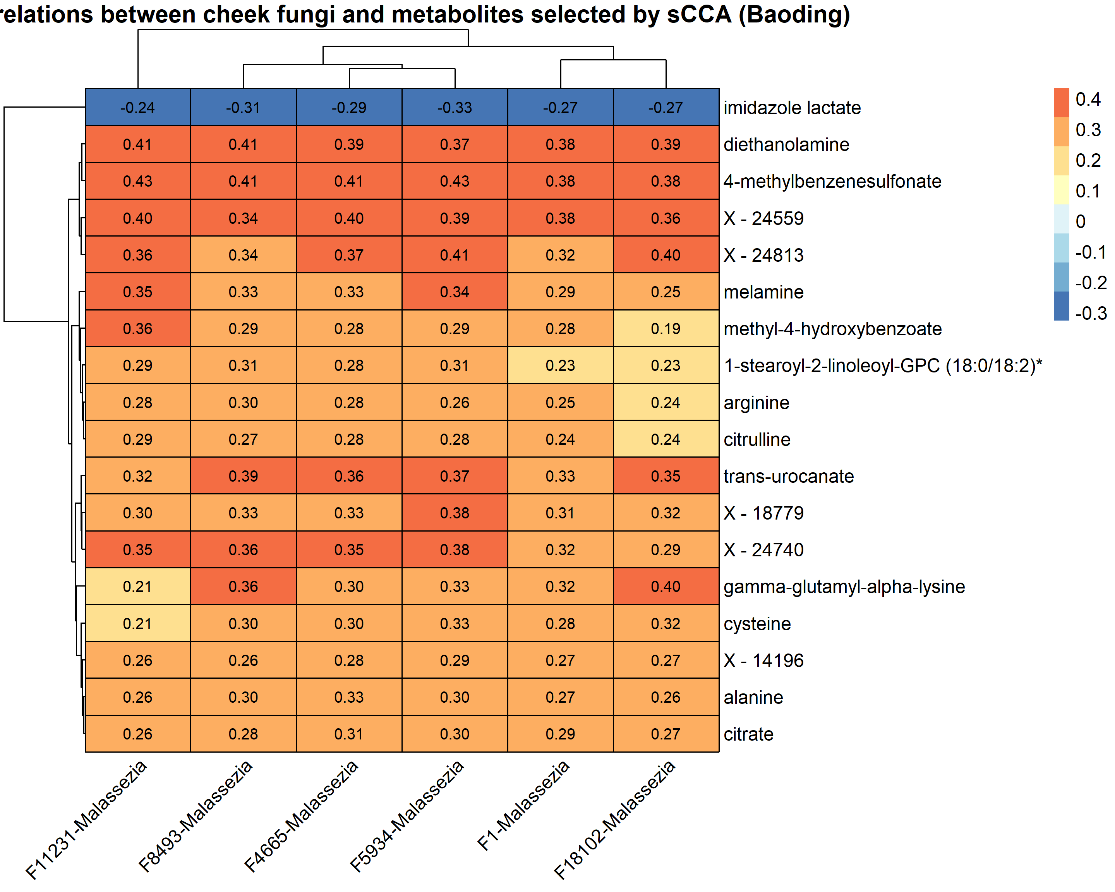
**

**Supplementary Figure 9**: Clinical study design, evaluation parameters, sampling and measurements. Baoding (More Polluted) and Dalian (Less Polluted) based on Air Quality Index (AQI).


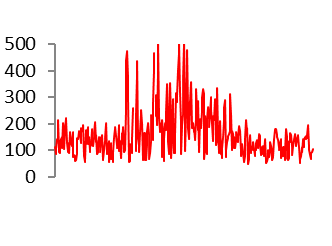


**AQI**


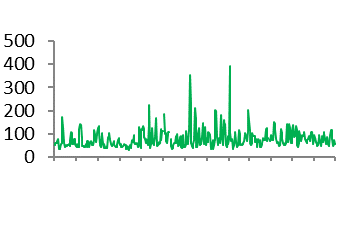


**375 Days**

**City 1: Baoding**

**City 2: Dalian**

AQI>100during 270 days

AQI>100during 83 days

n=67 Women (25-45yr)

n=67 Women (25-45yr)

**Clinical Evaluation**

**(Skin)**

**Biomonitoring**

**(Hair Shaft)**

**Biological Parameters**

**(Facial swabs & D-Squame)**

**Parameters**

**Measurements**

- **9 Clinical clusters**
- **35 Facial parameters**
- **15 PAH (Parent)**
- **56 Metabolites**

**(monohydroxy)**

- **350 Metabolites**
- **46 Proteins**
- **74 Skin Bacteria**
- **69 Skin Fungi**

**Study Population**
