# Supplementary material for: Multi-omics analysis to decipher the molecular link between chronic exposure to pollution and human skin dysfunction
Source: Sci Rep. 2021 Sep 15;11:18302. doi: 10.1038/s41598-021-97572-1 (PMC8443591; doi:10.1038/s41598-021-97572-1)
Supplement: Supplementary file 2 — Supplementary Table 1. [file 41598_2021_97572_MOESM2_ESM.docx]

**Figures and Tables**

**Supplementary Table 1: Clinical facial signs for all clinical signs, their evaluation across Cities and PAH levels**

PAH median corresponds to the median of the PAH score, the first component of a principal components analysis (PCA) on log-normalized PAH measurements. P-values were calculated using either unpaired two-samples Wilcoxon or chi² tests depending on whether the clinical scores were ordinal or binary.

| **CLINICAL SIGNS** | | **INTENSITY** | | **DALIAN** | **BAODING** | **P-value** | **PAH <MED** | **PAH >MED** | **P-value** |  |
| --- | --- | --- | --- | --- | --- | --- | --- | --- | --- | --- |
| **PIGMENTARY DISORDERS** |  | |  | |  |  |  |  |  | |
| **Spread macules on forehead** | | No | | 94.03 | 74.63 | 0.0029 | 91.04 | 77.61 | 0.0292 |  |
|  | | Weak | | 2.99 | 17.91 |  | 7.46 | 13.43 |  |  |
|  | | Moderate | | 2.99 | 2.99 |  | 1.49 | 4.48 |  |  |
|  | | Severe | |  | 4.48 |  |  | 4.48 |  |  |
| **Spread macules on cheeks** | | No | | 64.18 | 43.28 | 0.0024 | 61.19 | 46.27 | 0.0212 |  |
|  | | Weak | | 32.84 | 32.84 |  | 34.33 | 31.34 |  |  |
|  | | Moderate | | 2.99 | 19.40 |  | 2.99 | 19.40 |  |  |
|  | | Severe | |  | 4.48 |  | 1.49 | 2.99 |  |  |
| **Hyperpigmented spots on forehead** | | No | | 26.87 | 20.90 | 0.2844 | 25.37 | 22.39 | 0.4938 |  |
|  | | Weak | | 73.13 | 76.12 |  | 74.63 | 74.63 |  |  |
|  | | Moderate | |  | 2.99 |  |  | 2.99 |  |  |
| **Simplex lentigo on cheek** | | No | | 25.37 | 13.43 | 0.0805 | 25.37 | 13.43 | 0.0805 |  |
|  | | Yes | | 74.63 | 86.57 |  | 74.63 | 86.57 |  |  |
| **Actinic lentigines on cheek** | | No | | 77.61 | 76.12 | 0.8377 | 82.09 | 71.64 | 0.1516 |  |
|  | | Yes | | 22.39 | 23.88 |  | 17.91 | 28.36 |  |  |
| **Melasma or melasma like on forehead** | | No | | 97.01 | 91.04 | 0.1447 | 97.01 | 91.04 | 0.1447 |  |
|  | | Yes | | 2.99 | 8.96 |  | 2.99 | 8.96 |  |  |
| **Melasma or melasma like on cheek** | | No | | 80.60 | 85.07 | 0.4919 | 80.60 | 85.07 | 0.4919 |  |
|  | | Yes | | 19.40 | 14.93 |  | 19.40 | 14.93 |  |  |
| **WRINKLES** |  | |  | |  |  |  |  |  | |
| **Crow’s feet wrinkles** | | 0 | | 5.97 | 8.96 | 0.2761 | 5.97 | 8.96 | 0.6778 |  |
|  | | 1 | | 31.34 | 19.40 |  | 25.37 | 25.37 |  |  |
|  | | 2 | | 25.37 | 28.36 |  | 25.37 | 28.36 |  |  |
|  | | 3 | | 26.87 | 22.39 |  | 29.85 | 19.40 |  |  |
|  | | 4 | | 8.96 | 20.90 |  | 11.94 | 17.91 |  |  |
|  | | 5 | | 1.49 |  |  | 1.49 |  |  |  |
| **Glabellar wrinkles** | | 0 | | 16.42 | 23.88 | 0.6225 | 19.40 | 20.90 | 0.8282 |  |
|  | | 1 | | 62.69 | 43.28 |  | 55.22 | 50.75 |  |  |
|  | | 2 | | 19.40 | 28.36 |  | 23.88 | 23.88 |  |  |
|  | | 3 | | 1.49 | 4.48 |  | 1.49 | 4.48 |  |  |
| **Upper lips wrinkles** | | 0 | | 19.40 | 38.81 | 0.0182 | 25.37 | 32.84 | 0.2546 |  |
|  | | 1 | | 68.66 | 53.73 |  | 62.69 | 59.70 |  |  |
|  | | 2 | | 11.94 | 7.46 |  | 11.94 | 7.46 |  |  |
| **Forehead wrinkles** | | 0 | | 10.45 | 2.99 | 0.0072 | 10.45 | 2.99 | 0.0329 |  |
|  | | 1 | | 40.30 | 32.84 |  | 40.30 | 32.84 |  |  |
|  | | 2 | | 34.33 | 28.36 |  | 29.85 | 32.84 |  |  |
|  | | 3 | | 10.45 | 16.42 |  | 11.94 | 14.93 |  |  |
|  | | 4 | | 1.49 | 10.45 |  | 2.99 | 8.96 |  |  |
|  | | 5 | | 1.49 | 8.96 |  | 2.99 | 7.46 |  |  |
|  | | 6 | | 1.49 |  |  | 1.49 |  |  |  |
| **Ptosis skin laxity on lower face** | | No | | 14.93 | 10.45 | 0.4362 | 13.43 | 11.94 | 0.7952 |  |
|  | | Weak | | 85.07 | 89.55 |  | 86.57 | 88.06 |  |  |
| \| **WRINKLES** \| **ACNE** \|  \|  \|  \|  \|  \|  \| \| --- \| --- \| --- \| --- \| --- \| --- \| --- \| --- \| | | \| **WRINKLES** \|  \|  \|  \|  \|  \|  \|  \| \| --- \| --- \| --- \| --- \| --- \| --- \| --- \| --- \| | | \| **WRINKLES** \|  \|  \|  \|  \|  \|  \|  \| \| --- \| --- \| --- \| --- \| --- \| --- \| --- \| --- \| | \| **WRINKLES** \|  \|  \|  \|  \|  \|  \|  \| \| --- \| --- \| --- \| --- \| --- \| --- \| --- \| --- \| | \| **WRINKLES** \|  \|  \|  \|  \|  \|  \|  \| \| --- \| --- \| --- \| --- \| --- \| --- \| --- \| --- \| | \| **WRINKLES** \|  \|  \|  \|  \|  \|  \|  \| \| --- \| --- \| --- \| --- \| --- \| --- \| --- \| --- \| | \| **WRINKLES** \|  \|  \|  \|  \|  \|  \|  \| \| --- \| --- \| --- \| --- \| --- \| --- \| --- \| --- \| | \| **WRINKLES** \|  \|  \|  \|  \|  \|  \|  \| \| --- \| --- \| --- \| --- \| --- \| --- \| --- \| --- \| |  |
| **Is there any sign of acne in progress ?** | | No | | 49.25 | 38.81 | 0.2232 | 49.25 | 38.81 | 0.2232 |  |
|  | | Yes | | 50.75 | 61.19 |  | 50.75 | 61.19 |  |  |
| **Acne severity on the face** | | No | | 49.25 | 40.30 | 0.2198 | 49.25 | 40.30 | 0.2970 |  |
|  | | Weak | | 47.76 | 52.24 |  | 46.27 | 53.73 |  |  |
|  | | Moderate | | 2.99 | 7.46 |  | 4.48 | 5.97 |  |  |
| **Is there any pigmented acne** | | No | | 38.81 | 41.79 | 0.9002 | 40.30 | 40.30 | 0.8397 |  |
| **sequellae ?** | | Weak | | 59.70 | 53.73 |  | 55.22 | 58.21 |  |  |
|  | | Moderate | | 1.49 | 4.48 |  | 4.48 | 1.49 |  |  |
| **Is there any non-pigmented acne** | | No | | 56.72 | 47.76 | 0.1545 | 58.21 | 46.27 | 0.1142 |  |
| **scar ?** | | Weak | | 41.79 | 41.79 |  | 38.81 | 44.78 |  |  |
|  | | Moderate | | 1.49 | 8.96 |  | 2.99 | 7.46 |  |  |
|  | | Severe | |  | 1.49 |  |  | 1.49 |  |  |
| \| **WRINKLES** \| **PORE** \|  \|  \|  \|  \|  \|  \| \| --- \| --- \| --- \| --- \| --- \| --- \| --- \| --- \| | | \| **WRINKLES** \|  \|  \|  \|  \|  \|  \|  \| \| --- \| --- \| --- \| --- \| --- \| --- \| --- \| --- \| | | \| **WRINKLES** \|  \|  \|  \|  \|  \|  \|  \| \| --- \| --- \| --- \| --- \| --- \| --- \| --- \| --- \| | \| **WRINKLES** \|  \|  \|  \|  \|  \|  \|  \| \| --- \| --- \| --- \| --- \| --- \| --- \| --- \| --- \| | \| **WRINKLES** \|  \|  \|  \|  \|  \|  \|  \| \| --- \| --- \| --- \| --- \| --- \| --- \| --- \| --- \| | \| **WRINKLES** \|  \|  \|  \|  \|  \|  \|  \| \| --- \| --- \| --- \| --- \| --- \| --- \| --- \| --- \| | \| **WRINKLES** \|  \|  \|  \|  \|  \|  \|  \| \| --- \| --- \| --- \| --- \| --- \| --- \| --- \| --- \| | \| **WRINKLES** \|  \|  \|  \|  \|  \|  \|  \| \| --- \| --- \| --- \| --- \| --- \| --- \| --- \| --- \| |  |
| **Severity of facial pores on global face** | | No | | 2.99 | 7.46 | 0.0533 | 2.99 | 7.46 | 0.8899 |  |
|  | | Weak | | 82.09 | 58.21 |  | 74.63 | 65.67 |  |  |
|  | | Moderate | | 14.93 | 29.85 |  | 20.90 | 23.88 |  |  |
|  | | Severe | |  | 4.48 |  | 1.49 | 2.99 |  |  |
| \| **WRINKLES** \| **DRY** \|  \|  \|  \|  \|  \|  \| \| --- \| --- \| --- \| --- \| --- \| --- \| --- \| --- \| | | \| **WRINKLES** \|  \|  \|  \|  \|  \|  \|  \| \| --- \| --- \| --- \| --- \| --- \| --- \| --- \| --- \| | | \| **WRINKLES** \|  \|  \|  \|  \|  \|  \|  \| \| --- \| --- \| --- \| --- \| --- \| --- \| --- \| --- \| | \| **WRINKLES** \|  \|  \|  \|  \|  \|  \|  \| \| --- \| --- \| --- \| --- \| --- \| --- \| --- \| --- \| | \| **WRINKLES** \|  \|  \|  \|  \|  \|  \|  \| \| --- \| --- \| --- \| --- \| --- \| --- \| --- \| --- \| | \| **WRINKLES** \|  \|  \|  \|  \|  \|  \|  \| \| --- \| --- \| --- \| --- \| --- \| --- \| --- \| --- \| | \| **WRINKLES** \|  \|  \|  \|  \|  \|  \|  \| \| --- \| --- \| --- \| --- \| --- \| --- \| --- \| --- \| | \| **WRINKLES** \|  \|  \|  \|  \|  \|  \|  \| \| --- \| --- \| --- \| --- \| --- \| --- \| --- \| --- \| |  |
| **How dry is the subject skin on the** | | No | | 89.55 | 89.55 | 0.9799 | 86.57 | 92.54 | 0.2808 |  |
| **forehead nose and chin ?** | | Weaky | | 10.45 | 8.96 |  | 13.43 | 5.97 |  |  |
|  | | Moderately | |  | 1.49 |  | . | 1.49 |  |  |
| **How dry is the subject skin on the** | | No | | 91.04 | 92.54 | 0.7616 | 89.55 | 94.03 | 0.3585 |  |
| **Cheeks ?** | | Weaky | | 7.46 | 5.97 |  | 8.96 | 4.48 |  |  |
|  | | Moderately | | 1.49 | 1.49 |  | 1.49 | 1.49 |  |  |
| **How rough scaly by touching is the** | | No | | 86.57 | 82.09 | 0.4618 | 82.09 | 86.57 | 0.5079 |  |
| **subject skin on the forehead and nose ?** | | Weaky | | 13.43 | 16.42 |  | 17.91 | 11.94 |  |  |
|  | | Moderately | |  | 1.49 |  |  | 1.49 |  |  |
| **How rough scaly by touching is the** | | No | | 58.21 | 65.67 | 0.3270 | 58.21 | 65.67 | 0.3270. |  |
| **subject skin on the cheeks ?** | | Weaky | | 37.31 | 32.84 |  | 37.31 | 32.84 |  |  |
|  | | Moderately | | 4.48 | 1.49 |  | 4.48 | 1.49 |  |  |
| **Does the subject present dry patches** | | No | | 55.22 | 52.24 | 0.7290 | 52.24 | 55.22 | 0.7290 |  |
| **on the face ?** | | Yes | | 44.78 | 47.76 |  | 47.76 | 44.78 |  |  |
| \| **WRINKLES** \| **SHINY** \|  \|  \|  \|  \|  \|  \| \| --- \| --- \| --- \| --- \| --- \| --- \| --- \| --- \| | | \| **WRINKLES** \|  \|  \|  \|  \|  \|  \|  \| \| --- \| --- \| --- \| --- \| --- \| --- \| --- \| --- \| | | \| **WRINKLES** \|  \|  \|  \|  \|  \|  \|  \| \| --- \| --- \| --- \| --- \| --- \| --- \| --- \| --- \| | \| **WRINKLES** \|  \|  \|  \|  \|  \|  \|  \| \| --- \| --- \| --- \| --- \| --- \| --- \| --- \| --- \| | \| **WRINKLES** \|  \|  \|  \|  \|  \|  \|  \| \| --- \| --- \| --- \| --- \| --- \| --- \| --- \| --- \| | \| **WRINKLES** \|  \|  \|  \|  \|  \|  \|  \| \| --- \| --- \| --- \| --- \| --- \| --- \| --- \| --- \| | \| **WRINKLES** \|  \|  \|  \|  \|  \|  \|  \| \| --- \| --- \| --- \| --- \| --- \| --- \| --- \| --- \| | \| **WRINKLES** \|  \|  \|  \|  \|  \|  \|  \| \| --- \| --- \| --- \| --- \| --- \| --- \| --- \| --- \| |  |
|  | |  | |  |  |  |  |  |  |  |
| **How oily/greasy is the subjects skin** | | No | | 1.49 | 1.49 | 0.0086 | 2.99 |  | 0.0954 |  |
| **(by touching) on the forehead nose ?** | | Weaky | | 62.69 | 43.28 |  | 58.21 | 47.76 |  |  |
|  | | Moderately | | 31.34 | 34.33 |  | 28.36 | 37.31 |  |  |
|  | | Very | | 4.48 | 20.90 |  | 10.45 | 14.93 |  |  |
| **How oily/greasy is the subjects skin** | | No | | 83.58 | 65.67 | 0.0140 | 79.10 | 70.15 | 0.2160 |  |
| **(by touching) on the cheek ?** | | Weaky | | 16.42 | 28.36 |  | 19.40 | 25.37 |  |  |
|  | | Moderately | |  | 5.97 |  | 1.49 | 4.48 |  |  |
| **How shiny is the subjects skin** | | No | | 1.49 |  | 0.5365 | 1.49 |  | 0.8592 |  |
| **on the forehead nose and chin ?** | | Weaky | | 46.27 | 50.75 |  | 44.78 | 52.24 |  |  |
|  | | Moderately | | 50.75 | 32.84 |  | 47.76 | 35.82 |  |  |
|  | | Very | | 1.49 | 16.42 |  | 5.97 | 11.94 |  |  |
| **How shiny is the subjects skin** | | No | | 58.21 | 70.15 | 0.2627 | 56.72 | 71.64 | 0.1124 |  |
| **on the cheeks ?** | | Weaky | | 41.79 | 23.88 |  | 41.79 | 23.88 |  |  |
|  | | Moderately | |  | 5.97 |  | 1.49 | 4.48 |  |  |
| **EVEN** |  | |  | |  |  |  |  |  | |
| **How even is the subjects skin** | | Weaky | | 46.27 | 29.85 | 0.0520 | 49.25 | 26.87 | 0.0115 |  |
| **on the face ?** | | Moderately | | 46.27 | 58.21 |  | 43.28 | 61.19 |  |  |
|  | | Severe | | 7.46 | 11.94 |  | 7.46 | 11.94 |  |  |
| **UPPER EYELID** |  | |  | |  |  |  |  |  | |
| **Upper eyelid** | | No | | 64.18 | 65.67 | 0.8230 | 61.19 | 68.66 | 0.3632 |  |
|  | | Weaky | | 31.34 | 31.34 |  | 34.33 | 28.36 |  |  |
|  | | Moderate | | 4.48 | 1.49 |  | 4.48 | 1.49 |  |  |
|  | | Severe | |  | 1.49 |  |  | 1.49 |  |  |
| **SPOT DIAGNOSIS** |  | |  | |  |  |  |  |  | |
| **Probable spots diagnosis on cheeks :** | | No | | 19.40 | 17.91 | 0.8245 | 19.40 | 17.91 | 0.8245 |  |
| **Freckles** | | Yes | | 80.60 | 82.09 |  | 80.60 | 82.09 |  |  |
| **Probable spots diagnosis on cheek:** | | No | | 88.06 | 88.06 | 1.0000 | 88.06 | 88.06 | 1.0000 |  |
| **Seborrheric keratoses** | | Yes | | 11.94 | 11.94 |  | 11.94 | 11.94 |  |  |
| **Probable spots diagnosis on cheek:** | | No | | 40.30 | 43.28 | 0.7261 | 43.28 | 40.30 | 0.7261 |  |
| **Acne sequellae** | | Yes | | 59.70 | 56.72 |  | 56.72 | 59.70 |  |  |
| **Probable spots diagnosis on cheek:** | | No | | 4.48 | 8.96 | 0.3005 | 8.96 | 4.48 | 0.3005 |  |
| **Naevi** | | Yes | | 95.52 | 91.04 |  | 91.04 | 95.52 |  |  |
|  | |  | |  |  |  |  |  |  |  |
| **SKIN TONE HETEROGENEITY** |  | |  | |  |  |  |  |  | |
| **How dull is the subjects skin** | | No | | 100.0 | 91.04 | 0.0140 | 97.01 | 94.03 | 0.4033 |  |
| **on the face ?** | | Weaky | |  | 7.46 |  | 2.99 | 4.48 |  |  |
|  | | Moderately | |  | 1.49 |  |  | 1.49 |  |  |
| **Redness on the face** | | No | | 14.93 | 17.91 | 0.6454 | 14.93 | 17.91 | 0.6454 |  |
|  | | Weak | | 68.66 | 70.15 |  | 68.66 | 70.15 |  |  |
|  | | Moderately | | 14.93 | 7.46 |  | 14.93 | 7.46 |  |  |
|  | | Very | | 1.49 | 4.48 |  | 1.49 | 4.48 |  |  |
| **Blotchiness on the face** | | No | | 79.10 | 62.69 | 0.0364 | 76.12 | 65.67 | 0.1831 |  |
|  | | Yes | | 20.90 | 37.31 | . | 23.88 | 34.33 |  |  |
