# Supplementary material for: Multi-omics analysis to decipher the molecular link between chronic exposure to pollution and human skin dysfunction
Source: Sci Rep. 2021 Sep 15;11:18302. doi: 10.1038/s41598-021-97572-1 (PMC8443591; doi:10.1038/s41598-021-97572-1)
Supplement: Supplementary file 3 — Supplementary Table 2. [file 41598_2021_97572_MOESM3_ESM.docx]

**Supplementary Table 2:** List of metabolites with the pathway enrichment analysis. For each metabolite, we calculate the log2 Fold change Baoding/Dalian and we add the p-value and q-value. A red value indicates, for a given metabolite, that the difference in mean between the 2 cities is significant (q-value < 0.05, t-test with correction for multiple testing using Benjamini-Hochberg method).

|  | **Metabolites** | **Pathway** | **log2(FC)** | **p-value(FC)** | **q-value(FC)** |
| --- | --- | --- | --- | --- | --- |
| 1 | glycine | Amino Acid | 0.295 | 8.6e-03 | 3.9e-02 |
| 2 | N-acetylglycine | Amino Acid | 0.052 | 3.4e-01 | 4.9e-01 |
| 3 | sarcosine | Amino Acid | 0.873 | 1.4e-03 | 1.6e-02 |
| 4 | betaine | Amino Acid | 0.949 | 7.5e-02 | 1.6e-01 |
| 5 | serine | Amino Acid | 0.268 | 7.6e-03 | 3.7e-02 |
| 6 | N-acetylserine | Amino Acid | 0.145 | 6.2e-02 | 1.4e-01 |
| 7 | threonine | Amino Acid | 0.251 | 2.7e-02 | 7.5e-02 |
| 8 | N-acetylthreonine | Amino Acid | 0.170 | 1.5e-01 | 2.6e-01 |
| 9 | alanine | Amino Acid | 0.288 | 5.0e-03 | 3.1e-02 |
| 10 | N-acetylalanine | Amino Acid | 0.333 | 5.6e-03 | 3.1e-02 |
| 11 | aspartate | Amino Acid | 0.224 | 9.4e-03 | 4.0e-02 |
| 12 | N-acetylaspartate (NAA) | Amino Acid | 0.420 | 2.6e-02 | 7.4e-02 |
| 13 | asparagine | Amino Acid | 0.201 | 2.8e-02 | 7.8e-02 |
| 14 | glutamate | Amino Acid | 0.258 | 4.3e-03 | 2.9e-02 |
| 15 | glutamine | Amino Acid | 0.315 | 4.9e-03 | 3.1e-02 |
| 16 | N-acetylglutamate | Amino Acid | 0.144 | 3.3e-01 | 4.8e-01 |
| 17 | N-acetylglutamine | Amino Acid | -0.485 | 7.3e-01 | 8.1e-01 |
| 18 | glutamate, gamma-methyl ester | Amino Acid | -0.279 | 3.9e-01 | 5.2e-01 |
| 19 | pyroglutamine | Amino Acid | 0.770 | 4.6e-01 | 5.9e-01 |
| 20 | beta-citrylglutamate | Amino Acid | 0.281 | 1.9e-02 | 6.1e-02 |
| 21 | histidine | Amino Acid | 0.273 | 1.2e-02 | 4.7e-02 |
| 22 | N-acetylhistidine | Amino Acid | 0.211 | 1.5e-02 | 5.3e-02 |
| 23 | trans-urocanate | Amino Acid | 0.095 | 4.3e-01 | 5.6e-01 |
| 24 | cis-urocanate | Amino Acid | 0.361 | 2.0e-04 | 3.8e-03 |
| 25 | imidazole propionate | Amino Acid | 0.201 | 2.4e-01 | 3.8e-01 |
| 26 | imidazole lactate | Amino Acid | 0.501 | 6.1e-02 | 1.4e-01 |
| 27 | anserine | Amino Acid | -0.198 | 2.1e-01 | 3.5e-01 |
| 28 | histamine | Amino Acid | 0.785 | 9.3e-01 | 9.6e-01 |
| 29 | 4-imidazoleacetate | Amino Acid | 1.016 | 3.7e-07 | 6.2e-05 |
| 30 | lysine | Amino Acid | 0.030 | 5.4e-01 | 6.7e-01 |
| 31 | N6-acetyllysine | Amino Acid | -0.404 | 5.3e-01 | 6.6e-01 |
| 32 | N6,N6,N6-trimethyllysine | Amino Acid | -0.051 | 4.2e-01 | 5.5e-01 |
| 33 | phenylalanine | Amino Acid | 0.044 | 4.8e-01 | 6.2e-01 |
| 34 | N-acetylphenylalanine | Amino Acid | 0.182 | 2.5e-01 | 3.9e-01 |
| 35 | phenyllactate (PLA) | Amino Acid | -0.014 | 8.1e-01 | 8.9e-01 |
| 36 | tyrosine | Amino Acid | 0.070 | 3.0e-01 | 4.5e-01 |
| 37 | N-acetyltyrosine | Amino Acid | 0.208 | 1.8e-01 | 3.2e-01 |
| 38 | tyramine | Amino Acid | 0.002 | 4.1e-01 | 5.4e-01 |
| 39 | 4-hydroxyphenylpyruvate | Amino Acid | 0.387 | 5.9e-02 | 1.4e-01 |
| 40 | 3-(4-hydroxyphenyl)lactate | Amino Acid | -0.219 | 7.2e-01 | 8.0e-01 |
| 41 | phenol sulfate | Amino Acid | -0.255 | 1.0e-01 | 2.0e-01 |
| 42 | tryptophan | Amino Acid | 0.267 | 1.8e-02 | 6.0e-02 |
| 43 | kynurenine | Amino Acid | 0.034 | 2.1e-01 | 3.4e-01 |
| 44 | kynurenate | Amino Acid | 2.217 | 3.4e-05 | 1.1e-03 |
| 45 | 3-indoxyl sulfate | Amino Acid | -0.564 | 8.1e-03 | 3.8e-02 |
| 46 | leucine | Amino Acid | 0.181 | 7.3e-02 | 1.6e-01 |
| 47 | isovalerylglycine | Amino Acid | 0.276 | 3.1e-02 | 8.3e-02 |
| 48 | isovalerylcarnitine (C5) | Amino Acid | 0.336 | 1.3e-01 | 2.4e-01 |
| 49 | isoleucine | Amino Acid | 0.212 | 2.4e-02 | 6.9e-02 |
| 50 | ethylmalonate | Amino Acid | 0.784 | 1.3e-03 | 1.6e-02 |
| 51 | methylsuccinate | Amino Acid | 0.268 | 6.0e-03 | 3.2e-02 |
| 52 | valine | Amino Acid | 0.257 | 1.9e-02 | 6.1e-02 |
| 53 | N-acetylvaline | Amino Acid | 0.204 | 1.1e-01 | 2.2e-01 |
| 54 | 3-methyl-2-oxobutyrate | Amino Acid | 0.354 | 7.3e-02 | 1.6e-01 |
| 55 | methionine | Amino Acid | 0.076 | 4.0e-01 | 5.3e-01 |
| 56 | N-acetylmethionine | Amino Acid | -0.020 | 7.0e-01 | 8.0e-01 |
| 57 | methionine sulfoxide | Amino Acid | 0.149 | 8.2e-02 | 1.7e-01 |
| 58 | N-acetylmethionine sulfoxide | Amino Acid | 0.316 | 9.5e-02 | 1.9e-01 |
| 59 | cysteine | Amino Acid | -0.229 | 3.5e-01 | 5.0e-01 |
| 60 | cystine | Amino Acid | -0.084 | 8.1e-01 | 8.8e-01 |
| 61 | cysteine sulfinic acid | Amino Acid | -0.206 | 9.8e-01 | 9.8e-01 |
| 62 | taurine | Amino Acid | 0.202 | 1.3e-01 | 2.4e-01 |
| 63 | arginine | Amino Acid | 0.233 | 4.7e-02 | 1.2e-01 |
| 64 | argininosuccinate | Amino Acid | 0.243 | 7.9e-03 | 3.7e-02 |
| 65 | urea | Amino Acid | -0.172 | 7.0e-01 | 8.0e-01 |
| 66 | ornithine | Amino Acid | 0.272 | 2.1e-02 | 6.5e-02 |
| 67 | 2-oxoarginine | Amino Acid | 1.095 | 1.0e-03 | 1.4e-02 |
| 68 | citrulline | Amino Acid | 0.192 | 6.1e-02 | 1.4e-01 |
| 69 | proline | Amino Acid | 0.248 | 1.1e-02 | 4.6e-02 |
| 70 | dimethylarginine (SDMA + ADMA) | Amino Acid | 0.042 | 5.2e-01 | 6.5e-01 |
| 71 | N-acetylarginine | Amino Acid | 1.061 | 3.0e-06 | 1.7e-04 |
| 72 | N-delta-acetylornithine | Amino Acid | -0.209 | 9.5e-01 | 9.7e-01 |
| 73 | trans-4-hydroxyproline | Amino Acid | -0.780 | 1.4e-03 | 1.6e-02 |
| 74 | N-monomethylarginine | Amino Acid | 0.019 | 5.1e-01 | 6.4e-01 |
| 75 | guanidinoacetate | Amino Acid | 0.438 | 5.8e-03 | 3.2e-02 |
| 76 | creatine | Amino Acid | -0.043 | 8.9e-01 | 9.4e-01 |
| 77 | creatinine | Amino Acid | 0.117 | 5.5e-01 | 6.8e-01 |
| 78 | spermidine | Amino Acid | 0.148 | 9.2e-01 | 9.6e-01 |
| 79 | acisoga | Amino Acid | 0.613 | 1.4e-02 | 5.3e-02 |
| 80 | 4-guanidinobutanoate | Amino Acid | 0.801 | 4.8e-05 | 1.3e-03 |
| 81 | 5-oxoproline | Amino Acid | 0.263 | 4.7e-03 | 3.0e-02 |
| 82 | 2-hydroxybutyrate/2-hydroxyisobutyrate | Amino Acid | -0.049 | 3.8e-01 | 5.2e-01 |
| 83 | gamma-glutamylalanine | Peptide | 0.300 | 5.4e-03 | 3.1e-02 |
| 84 | gamma-glutamylglutamate | Peptide | 0.896 | 5.6e-04 | 9.9e-03 |
| 85 | gamma-glutamylglutamine | Peptide | 0.359 | 6.0e-04 | 9.9e-03 |
| 86 | gamma-glutamylglycine | Peptide | 0.379 | 3.8e-03 | 2.7e-02 |
| 87 | gamma-glutamylhistidine | Peptide | 0.268 | 1.7e-02 | 5.8e-02 |
| 88 | gamma-glutamylisoleucine | Peptide | 0.321 | 4.3e-03 | 2.9e-02 |
| 89 | gamma-glutamylleucine | Peptide | 0.415 | 1.3e-03 | 1.6e-02 |
| 90 | gamma-glutamyl-alpha-lysine | Peptide | 0.020 | 6.4e-01 | 7.6e-01 |
| 91 | gamma-glutamyl-epsilon-lysine | Peptide | -0.036 | 7.1e-01 | 8.0e-01 |
| 92 | gamma-glutamylmethionine | Peptide | 0.286 | 7.6e-02 | 1.6e-01 |
| 93 | gamma-glutamylphenylalanine | Peptide | 0.288 | 1.3e-02 | 5.1e-02 |
| 94 | gamma-glutamylthreonine | Peptide | 0.326 | 6.3e-03 | 3.3e-02 |
| 95 | gamma-glutamyltryptophan | Peptide | 0.357 | 4.4e-03 | 2.9e-02 |
| 96 | gamma-glutamyltyrosine | Peptide | 0.243 | 2.9e-02 | 7.9e-02 |
| 97 | gamma-glutamylvaline | Peptide | 0.335 | 2.2e-03 | 2.0e-02 |
| 98 | gamma-glutamylserine | Peptide | 0.402 | 1.6e-03 | 1.6e-02 |
| 99 | glycylisoleucine | Peptide | 0.207 | 8.1e-02 | 1.7e-01 |
| 100 | glycylleucine | Peptide | 0.160 | 5.3e-01 | 6.6e-01 |
| 101 | glycylvaline | Peptide | 0.183 | 8.5e-02 | 1.8e-01 |
| 102 | isoleucylglycine | Peptide | 0.168 | 1.0e-01 | 2.0e-01 |
| 103 | leucylalanine | Peptide | 0.106 | 4.2e-01 | 5.5e-01 |
| 104 | leucylglycine | Peptide | 0.122 | 1.0e-01 | 2.0e-01 |
| 105 | phenylalanylglycine | Peptide | 0.080 | 3.8e-01 | 5.2e-01 |
| 106 | prolylglycine | Peptide | 0.023 | 3.8e-01 | 5.2e-01 |
| 107 | tryptophylglycine | Peptide | 0.290 | 1.2e-01 | 2.3e-01 |
| 108 | tyrosylglycine | Peptide | 0.011 | 5.4e-01 | 6.7e-01 |
| 109 | valylglutamine | Peptide | 0.332 | 5.9e-03 | 3.2e-02 |
| 110 | valylglycine | Peptide | 0.210 | 5.0e-02 | 1.2e-01 |
| 111 | valylleucine | Peptide | -0.077 | 8.6e-01 | 9.2e-01 |
| 112 | leucylglutamine* | Peptide | 0.428 | 1.1e-02 | 4.6e-02 |
| 113 | phenylacetylglutamine | Peptide | -0.463 | 2.8e-02 | 7.8e-02 |
| 114 | glucose | Carbohydrate | -0.299 | 7.2e-01 | 8.0e-01 |
| 115 | pyruvate | Carbohydrate | 0.135 | 2.9e-01 | 4.4e-01 |
| 116 | lactate | Carbohydrate | 0.196 | 3.9e-02 | 1.0e-01 |
| 117 | glycerate | Carbohydrate | 0.031 | 3.6e-01 | 5.1e-01 |
| 118 | ribonate | Carbohydrate | 0.610 | 2.4e-01 | 3.8e-01 |
| 119 | arabitol/xylitol | Carbohydrate | 0.132 | 1.5e-01 | 2.6e-01 |
| 120 | arabonate/xylonate | Carbohydrate | -0.205 | 6.8e-01 | 7.9e-01 |
| 121 | maltose | Carbohydrate | 1.054 | 2.9e-03 | 2.4e-02 |
| 122 | sucrose | Carbohydrate | -0.741 | 3.5e-01 | 5.0e-01 |
| 123 | fructose | Carbohydrate | -0.420 | 6.3e-01 | 7.5e-01 |
| 124 | mannitol/sorbitol | Carbohydrate | -0.256 | 5.9e-01 | 7.2e-01 |
| 125 | mannose | Carbohydrate | 0.226 | 1.9e-01 | 3.2e-01 |
| 126 | N-acetylneuraminate | Carbohydrate | -0.243 | 9.6e-01 | 9.8e-01 |
| 127 | erythronate* | Carbohydrate | -0.386 | 6.3e-01 | 7.4e-01 |
| 128 | citrate | Energy | -0.147 | 3.1e-01 | 4.6e-01 |
| 129 | aconitate [cis or trans] | Energy | 0.197 | 7.2e-02 | 1.6e-01 |
| 130 | alpha-ketoglutarate | Energy | 0.342 | 4.5e-02 | 1.1e-01 |
| 131 | succinate | Energy | 0.225 | 4.2e-02 | 1.1e-01 |
| 132 | fumarate | Energy | 0.242 | 1.7e-02 | 5.8e-02 |
| 133 | malate | Energy | 0.056 | 1.7e-02 | 5.8e-02 |
| 134 | citraconate/glutaconate | Energy | 0.233 | 1.3e-02 | 5.1e-02 |
| 135 | malonate | Lipid | 0.300 | 7.6e-04 | 1.2e-02 |
| 136 | laurate (12:0) | Lipid | 0.900 | 4.9e-06 | 2.4e-04 |
| 137 | myristate (14:0) | Lipid | 0.375 | 1.4e-03 | 1.6e-02 |
| 138 | myristoleate (14:1n5) | Lipid | 0.138 | 5.2e-02 | 1.2e-01 |
| 139 | pentadecanoate (15:0) | Lipid | 0.386 | 7.9e-03 | 3.7e-02 |
| 140 | palmitate (16:0) | Lipid | 0.377 | 5.7e-04 | 9.9e-03 |
| 141 | margarate (17:0) | Lipid | 0.279 | 1.1e-02 | 4.6e-02 |
| 142 | 10-heptadecenoate (17:1n7) | Lipid | 0.260 | 1.4e-02 | 5.3e-02 |
| 143 | stearate (18:0) | Lipid | 0.279 | 5.5e-03 | 3.1e-02 |
| 144 | oleate/vaccenate (18:1) | Lipid | 0.508 | 4.2e-05 | 1.2e-03 |
| 145 | nonadecanoate (19:0) | Lipid | 0.251 | 1.9e-02 | 6.1e-02 |
| 146 | 10-nonadecenoate (19:1n9) | Lipid | 0.366 | 9.0e-03 | 3.9e-02 |
| 147 | arachidate (20:0) | Lipid | 0.226 | 1.3e-02 | 5.1e-02 |
| 148 | eicosenoate (20:1) | Lipid | 0.256 | 3.7e-03 | 2.7e-02 |
| 149 | erucate (22:1n9) | Lipid | 0.288 | 8.4e-03 | 3.8e-02 |
| 150 | docosapentaenoate (n3 DPA; 22:5n3) | Lipid | 0.022 | 1.5e-01 | 2.6e-01 |
| 151 | linoleate (18:2n6) | Lipid | 0.704 | 2.0e-06 | 1.4e-04 |
| 152 | linolenate [alpha or gamma; (18:3n3 or 6)] | Lipid | 0.446 | 1.0e-04 | 2.6e-03 |
| 153 | dihomo-linolenate (20:3n3 or n6) | Lipid | 0.191 | 2.1e-02 | 6.4e-02 |
| 154 | arachidonate (20:4n6) | Lipid | 0.271 | 5.2e-03 | 3.1e-02 |
| 155 | docosadienoate (22:2n6) | Lipid | 0.371 | 3.8e-03 | 2.7e-02 |
| 156 | linoelaidate (tr 18:2n6) | Lipid | 0.268 | 6.1e-02 | 1.4e-01 |
| 157 | mead acid (20:3n9) | Lipid | 0.181 | 7.4e-03 | 3.7e-02 |
| 158 | (12 or 13)-methylmyristate (a15:0 or i15:0) | Lipid | 0.405 | 2.0e-03 | 2.0e-02 |
| 159 | (14 or 15)-methylpalmitate (a17:0 or i17:0) | Lipid | 0.455 | 3.5e-03 | 2.7e-02 |
| 160 | (16 or 17)-methylstearate (a19:0 or i19:0) | Lipid | 0.356 | 1.9e-02 | 6.1e-02 |
| 161 | dimethylmalonic acid | Lipid | 0.174 | 1.3e-01 | 2.4e-01 |
| 162 | glutarate (C5-DC) | Lipid | 0.155 | 4.5e-02 | 1.1e-01 |
| 163 | 3-methylglutarate/2-methylglutarate | Lipid | 0.170 | 2.1e-02 | 6.4e-02 |
| 164 | adipate (C6-DC) | Lipid | 0.160 | 5.9e-03 | 3.2e-02 |
| 165 | 2-hydroxyadipate | Lipid | 0.285 | 1.5e-02 | 5.3e-02 |
| 166 | maleate | Lipid | 0.706 | 1.5e-06 | 1.3e-04 |
| 167 | pimelate (C7-DC) | Lipid | 0.141 | 2.5e-02 | 7.2e-02 |
| 168 | suberate (C8-DC) | Lipid | 0.407 | 1.6e-03 | 1.6e-02 |
| 169 | azelate (C9-DC) | Lipid | 0.194 | 2.9e-02 | 8.0e-02 |
| 170 | sebacate (C10-DC) | Lipid | 0.276 | 2.5e-03 | 2.2e-02 |
| 171 | undecanedioate (C11-DC) | Lipid | 0.186 | 2.0e-02 | 6.1e-02 |
| 172 | dodecanedioate (C12-DC) | Lipid | 0.310 | 8.8e-03 | 3.9e-02 |
| 173 | tetradecanedioate (C14-DC) | Lipid | 0.195 | 8.8e-02 | 1.8e-01 |
| 174 | oleamide | Lipid | 0.611 | 3.1e-05 | 1.1e-03 |
| 175 | palmitamide (16:0) | Lipid | 0.580 | 8.4e-06 | 3.7e-04 |
| 176 | methylmalonate (MMA) | Lipid | 0.486 | 1.6e-02 | 5.5e-02 |
| 177 | N-octanoylglycine | Lipid | -2.064 | 3.3e-01 | 4.8e-01 |
| 178 | N-palmitoylglycine | Lipid | -0.256 | 6.5e-01 | 7.6e-01 |
| 179 | octanoylcarnitine (C8) | Lipid | 0.240 | 8.7e-03 | 3.9e-02 |
| 180 | laurylcarnitine (C12) | Lipid | 0.206 | 1.9e-01 | 3.2e-01 |
| 181 | myristoylcarnitine (C14) | Lipid | -0.040 | 6.2e-01 | 7.4e-01 |
| 182 | palmitoylcarnitine (C16) | Lipid | 0.321 | 1.2e-02 | 4.8e-02 |
| 183 | palmitoleoylcarnitine (C16:1)* | Lipid | 0.176 | 4.5e-01 | 5.9e-01 |
| 184 | stearoylcarnitine (C18) | Lipid | 0.191 | 1.1e-01 | 2.2e-01 |
| 185 | oleoylcarnitine (C18:1) | Lipid | 0.296 | 1.9e-01 | 3.2e-01 |
| 186 | myristoleoylcarnitine (C14:1)* | Lipid | 0.118 | 9.8e-01 | 9.8e-01 |
| 187 | carnitine | Lipid | -0.101 | 6.9e-01 | 8.0e-01 |
| 188 | 2-hydroxydecanoate | Lipid | 0.366 | 6.9e-03 | 3.5e-02 |
| 189 | 2-hydroxymyristate | Lipid | -0.034 | 1.1e-01 | 2.1e-01 |
| 190 | 2-hydroxypalmitate | Lipid | -0.022 | 7.3e-02 | 1.6e-01 |
| 191 | 2-hydroxystearate | Lipid | -0.092 | 1.3e-01 | 2.4e-01 |
| 192 | 3-hydroxylaurate | Lipid | 0.693 | 1.7e-03 | 1.7e-02 |
| 193 | 3-hydroxymyristate | Lipid | 0.563 | 2.4e-03 | 2.1e-02 |
| 194 | 3-hydroxypalmitate | Lipid | 0.168 | 4.3e-02 | 1.1e-01 |
| 195 | 13-HODE + 9-HODE | Lipid | 0.163 | 9.1e-02 | 1.8e-01 |
| 196 | 3-hydroxystearate | Lipid | 0.027 | 2.1e-01 | 3.5e-01 |
| 197 | oleoyl ethanolamide | Lipid | 0.602 | 3.0e-03 | 2.4e-02 |
| 198 | myristoyl ethanolamide | Lipid | 0.506 | 1.1e-01 | 2.1e-01 |
| 199 | palmitoyl ethanolamide | Lipid | 0.357 | 7.6e-03 | 3.7e-02 |
| 200 | stearoyl ethanolamide | Lipid | 0.265 | 3.4e-03 | 2.7e-02 |
| 201 | linoleoyl ethanolamide | Lipid | 0.910 | 5.3e-07 | 6.2e-05 |
| 202 | myo-inositol | Lipid | -0.275 | 6.6e-01 | 7.7e-01 |
| 203 | choline | Lipid | 0.198 | 9.2e-02 | 1.9e-01 |
| 204 | glycerophosphorylcholine (GPC) | Lipid | -0.234 | 8.3e-01 | 9.0e-01 |
| 205 | 1-palmitoyl-2-oleoyl-GPC (16:0/18:1) | Lipid | -0.476 | 1.6e-01 | 2.8e-01 |
| 206 | 1-palmitoyl-2-linoleoyl-GPC (16:0/18:2) | Lipid | -0.564 | 5.9e-02 | 1.4e-01 |
| 207 | 1-stearoyl-2-linoleoyl-GPC (18:0/18:2)* | Lipid | -0.209 | 8.7e-01 | 9.3e-01 |
| 208 | glycerol | Lipid | 0.201 | 2.4e-01 | 3.8e-01 |
| 209 | glycerol 3-phosphate | Lipid | 0.295 | 1.3e-04 | 2.9e-03 |
| 210 | glycerophosphoglycerol | Lipid | 0.130 | 2.6e-01 | 4.1e-01 |
| 211 | 1-myristoylglycerol (14:0) | Lipid | 0.334 | 7.0e-03 | 3.5e-02 |
| 212 | 1-pentadecanoylglycerol (15:0) | Lipid | 0.329 | 1.4e-02 | 5.3e-02 |
| 213 | 1-palmitoylglycerol (16:0) | Lipid | 0.206 | 4.4e-03 | 2.9e-02 |
| 214 | 1-palmitoleoylglycerol (16:1)* | Lipid | 0.337 | 2.3e-03 | 2.1e-02 |
| 215 | 1-margaroylglycerol (17:0) | Lipid | 0.442 | 1.4e-02 | 5.3e-02 |
| 216 | 1-oleoylglycerol (18:1) | Lipid | 0.434 | 7.4e-04 | 1.2e-02 |
| 217 | 1-linoleoylglycerol (18:2) | Lipid | 0.479 | 9.6e-04 | 1.3e-02 |
| 218 | 2-myristoylglycerol (14:0) | Lipid | 0.492 | 3.1e-03 | 2.4e-02 |
| 219 | 2-palmitoylglycerol (16:0) | Lipid | 0.211 | 5.4e-03 | 3.1e-02 |
| 220 | 2-oleoylglycerol (18:1) | Lipid | 0.450 | 2.8e-03 | 2.4e-02 |
| 221 | oleoyl-linoleoyl-glycerol (18:1/18:2) [1] | Lipid | -0.135 | 6.2e-01 | 7.4e-01 |
| 222 | oleoyl-linoleoyl-glycerol (18:1/18:2) [2] | Lipid | -0.291 | 3.9e-01 | 5.2e-01 |
| 223 | linoleoyl-linoleoyl-glycerol (18:2/18:2) [1]* | Lipid | 0.259 | 6.9e-02 | 1.5e-01 |
| 224 | linoleoyl-linolenoyl-glycerol (18:2/18:3) [2]* | Lipid | -0.101 | 7.2e-01 | 8.0e-01 |
| 225 | sphinganine | Lipid | -0.037 | 5.7e-01 | 7.0e-01 |
| 226 | phytosphingosine | Lipid | -0.072 | 9.1e-01 | 9.5e-01 |
| 227 | N-palmitoyl-sphinganine (d18:0/16:0) | Lipid | -0.038 | 7.0e-01 | 8.0e-01 |
| 228 | N-palmitoyl-sphingosine (d18:1/16:0) | Lipid | 0.011 | 6.2e-01 | 7.4e-01 |
| 229 | N-stearoyl-sphingosine (d18:1/18:0)* | Lipid | -0.085 | 9.5e-01 | 9.8e-01 |
| 230 | palmitoyl sphingomyelin (d18:1/16:0) | Lipid | -0.554 | 4.7e-02 | 1.2e-01 |
| 231 | sphingosine | Lipid | -0.047 | 6.9e-01 | 8.0e-01 |
| 232 | 3-hydroxy-3-methylglutarate | Lipid | 0.459 | 1.2e-04 | 2.7e-03 |
| 233 | cholesterol | Lipid | 0.068 | 3.2e-01 | 4.7e-01 |
| 234 | 4-cholesten-3-one | Lipid | 1.032 | 1.7e-02 | 5.8e-02 |
| 235 | 7-hydroxycholesterol (alpha or beta) | Lipid | 0.060 | 9.9e-02 | 2.0e-01 |
| 236 | inosine | Nucleotide | 0.044 | 7.2e-01 | 8.0e-01 |
| 237 | hypoxanthine | Nucleotide | 0.148 | 2.8e-01 | 4.3e-01 |
| 238 | xanthine | Nucleotide | 0.199 | 1.0e-01 | 2.0e-01 |
| 239 | xanthosine | Nucleotide | -0.099 | 2.4e-01 | 3.8e-01 |
| 240 | 2'-deoxyinosine | Nucleotide | 0.329 | 8.6e-02 | 1.8e-01 |
| 241 | urate | Nucleotide | 0.049 | 9.6e-01 | 9.8e-01 |
| 242 | allantoin | Nucleotide | -0.293 | 2.3e-01 | 3.7e-01 |
| 243 | adenosine | Nucleotide | 0.201 | 3.7e-02 | 9.7e-02 |
| 244 | adenine | Nucleotide | 0.113 | 1.8e-01 | 3.2e-01 |
| 245 | guanosine | Nucleotide | -0.022 | 7.2e-01 | 8.0e-01 |
| 246 | guanine | Nucleotide | -0.879 | 5.3e-03 | 3.1e-02 |
| 247 | orotate | Nucleotide | 0.242 | 1.3e-01 | 2.4e-01 |
| 248 | uridine | Nucleotide | 0.183 | 1.8e-01 | 3.1e-01 |
| 249 | uracil | Nucleotide | 0.165 | 2.1e-01 | 3.5e-01 |
| 250 | pseudouridine | Nucleotide | 0.012 | 8.5e-01 | 9.1e-01 |
| 251 | 2'-deoxyuridine | Nucleotide | 0.079 | 3.5e-01 | 5.0e-01 |
| 252 | beta-alanine | Nucleotide | 0.013 | 8.7e-01 | 9.3e-01 |
| 253 | cytidine | Nucleotide | -0.084 | 1.2e-01 | 2.3e-01 |
| 254 | 2'-deoxycytidine | Nucleotide | 0.237 | 1.9e-01 | 3.2e-01 |
| 255 | thymine | Nucleotide | 0.295 | 1.3e-01 | 2.4e-01 |
| 256 | nicotinamide | Cofactors and Vitamins | 1.996 | 5.6e-02 | 1.3e-01 |
| 257 | nicotinamide adenine dinucleotide (NAD+) | Cofactors and Vitamins | 0.179 | 9.0e-02 | 1.8e-01 |
| 258 | trigonelline (N'-methylnicotinate) | Cofactors and Vitamins | 0.206 | 9.7e-02 | 1.9e-01 |
| 259 | N1-Methyl-2-pyridone-5-carboxamide | Cofactors and Vitamins | -0.224 | 3.2e-01 | 4.7e-01 |
| 260 | pantothenate | Cofactors and Vitamins | 0.568 | 1.2e-01 | 2.3e-01 |
| 261 | threonate | Cofactors and Vitamins | -0.104 | 3.7e-01 | 5.1e-01 |
| 262 | alpha-tocopherol | Cofactors and Vitamins | 0.032 | 2.8e-01 | 4.2e-01 |
| 263 | alpha-tocopherol acetate | Cofactors and Vitamins | -0.177 | 9.6e-01 | 9.8e-01 |
| 264 | pyridoxate | Cofactors and Vitamins | 0.122 | 6.3e-01 | 7.4e-01 |
| 265 | hippurate | Xenobiotics | -0.637 | 1.9e-02 | 6.1e-02 |
| 266 | 4-hydroxybenzoate | Xenobiotics | -0.610 | 9.6e-01 | 9.8e-01 |
| 267 | methyl-4-hydroxybenzoate | Xenobiotics | -1.178 | 4.9e-01 | 6.2e-01 |
| 268 | propyl 4-hydroxybenzoate | Xenobiotics | -0.901 | 9.3e-01 | 9.7e-01 |
| 269 | p-cresol sulfate | Xenobiotics | -1.324 | 8.6e-04 | 1.2e-02 |
| 270 | theobromine | Xenobiotics | -1.402 | 9.9e-06 | 3.8e-04 |
| 271 | nicotine | Xenobiotics | -1.854 | 5.8e-02 | 1.4e-01 |
| 272 | gluconate | Xenobiotics | 0.290 | 4.3e-02 | 1.1e-01 |
| 273 | erythritol | Xenobiotics | 0.161 | 7.3e-01 | 8.1e-01 |
| 274 | hydroquinone beta-D-glucopyranoside | Xenobiotics | -0.622 | 2.6e-02 | 7.4e-02 |
| 275 | piperine | Xenobiotics | -1.359 | 7.0e-02 | 1.5e-01 |
| 276 | quinate | Xenobiotics | -0.922 | 5.8e-01 | 7.0e-01 |
| 277 | saccharin | Xenobiotics | -0.042 | 9.5e-01 | 9.7e-01 |
| 278 | acesulfame | Xenobiotics | 1.378 | 3.4e-02 | 8.9e-02 |
| 279 | stachydrine | Xenobiotics | -0.553 | 5.1e-02 | 1.2e-01 |
| 280 | tartarate | Xenobiotics | 0.345 | 1.8e-02 | 5.9e-02 |
| 281 | methyl glucopyranoside (alpha + beta) | Xenobiotics | -0.067 | 8.1e-01 | 8.9e-01 |
| 282 | salicylate | Xenobiotics | 0.461 | 1.7e-04 | 3.6e-03 |
| 283 | 2-dimethylaminoethanol | Xenobiotics | -0.309 | 2.0e-01 | 3.2e-01 |
| 284 | diethanolamine | Xenobiotics | 0.102 | 4.0e-01 | 5.3e-01 |
| 285 | diglycerol | Xenobiotics | -0.149 | 5.0e-01 | 6.3e-01 |
| 286 | lauryl sulfate | Xenobiotics | 0.120 | 1.9e-01 | 3.2e-01 |
| 287 | myristoyl sulfate | Xenobiotics | 0.062 | 2.5e-01 | 3.9e-01 |
| 288 | dexpanthenol | Xenobiotics | -1.071 | 2.3e-01 | 3.7e-01 |
| 289 | melamine | Xenobiotics | -0.194 | 2.4e-01 | 3.8e-01 |
| 290 | triethanolamine | Xenobiotics | 0.036 | 2.4e-01 | 3.8e-01 |
| 291 | 4-methylbenzenesulfonate | Xenobiotics | -0.162 | 2.0e-01 | 3.4e-01 |
| 292 | thioproline | Xenobiotics | -0.520 | 1.5e-02 | 5.3e-02 |
| 293 | X - 11612 | Unknown | -0.040 | 3.6e-01 | 5.0e-01 |
| 294 | X - 12100 | Unknown | 0.333 | 3.1e-02 | 8.3e-02 |
| 295 | X - 12565 | Unknown | 0.107 | 3.8e-01 | 5.2e-01 |
| 296 | X - 13007 | Unknown | 0.020 | 1.0e+00 | 1.0e+00 |
| 297 | X - 13504 | Unknown | 0.142 | 3.8e-01 | 5.2e-01 |
| 298 | X - 13737 | Unknown | 2.038 | 2.9e-15 | 1.0e-12 |
| 299 | X - 14056 | Unknown | -0.099 | 5.0e-01 | 6.3e-01 |
| 300 | X - 14095 | Unknown | 0.253 | 5.5e-02 | 1.3e-01 |
| 301 | X - 14096 | Unknown | 0.111 | 3.0e-01 | 4.5e-01 |
| 302 | X - 14099 | Unknown | 0.177 | 1.4e-01 | 2.6e-01 |
| 303 | X - 14113 | Unknown | 0.314 | 2.3e-02 | 6.9e-02 |
| 304 | X - 14141 | Unknown | 0.189 | 1.3e-01 | 2.3e-01 |
| 305 | X - 14196 | Unknown | -0.047 | 9.9e-01 | 9.9e-01 |
| 306 | X - 14264 | Unknown | 0.212 | 4.9e-01 | 6.2e-01 |
| 307 | X - 14302 | Unknown | 0.051 | 3.9e-01 | 5.2e-01 |
| 308 | X - 14314 | Unknown | 0.121 | 1.5e-01 | 2.6e-01 |
| 309 | X - 14364 | Unknown | -0.093 | 8.9e-01 | 9.4e-01 |
| 310 | X - 14697 | Unknown | 0.252 | 3.2e-01 | 4.7e-01 |
| 311 | X - 14900 | Unknown | -0.009 | 9.0e-01 | 9.5e-01 |
| 312 | X - 14904 | Unknown | 0.137 | 3.1e-01 | 4.6e-01 |
| 313 | X - 15497 | Unknown | -0.195 | 8.0e-01 | 8.8e-01 |
| 314 | X - 15666 | Unknown | 0.056 | 2.7e-01 | 4.2e-01 |
| 315 | X - 16267 | Unknown | 0.442 | 3.2e-02 | 8.5e-02 |
| 316 | X - 16938 | Unknown | 0.233 | 3.8e-02 | 9.9e-02 |
| 317 | X - 17325 | Unknown | -0.139 | 6.6e-01 | 7.7e-01 |
| 318 | X - 17801 | Unknown | 0.119 | 3.4e-01 | 4.8e-01 |
| 319 | X - 17807 | Unknown | -0.094 | 8.3e-01 | 9.0e-01 |
| 320 | X - 17825 | Unknown | 0.310 | 1.0e-02 | 4.3e-02 |
| 321 | X - 17842 | Unknown | 0.235 | 6.4e-02 | 1.4e-01 |
| 322 | X - 17855 | Unknown | 0.115 | 6.0e-02 | 1.4e-01 |
| 323 | X - 18165 | Unknown | -0.068 | 9.2e-01 | 9.6e-01 |
| 324 | X - 18779 | Unknown | -0.098 | 4.5e-01 | 5.9e-01 |
| 325 | X - 18889 | Unknown | -0.038 | 6.9e-01 | 8.0e-01 |
| 326 | X - 19299 | Unknown | 0.331 | 2.9e-01 | 4.4e-01 |
| 327 | X - 19561 | Unknown | -0.181 | 6.9e-01 | 8.0e-01 |
| 328 | X - 19932 | Unknown | 0.133 | 1.8e-01 | 3.2e-01 |
| 329 | X - 22035 | Unknown | -0.060 | 4.9e-01 | 6.2e-01 |
| 330 | X - 22102 | Unknown | 0.130 | 4.4e-01 | 5.7e-01 |
| 331 | X - 22800 | Unknown | 0.874 | 1.6e-02 | 5.5e-02 |
| 332 | X - 23196 | Unknown | -0.003 | 3.0e-01 | 4.5e-01 |
| 333 | X - 23240 | Unknown | 0.318 | 1.5e-02 | 5.3e-02 |
| 334 | X - 23277 | Unknown | 0.047 | 6.1e-01 | 7.3e-01 |
| 335 | X - 23469 | Unknown | -0.195 | 8.8e-01 | 9.3e-01 |
| 336 | X - 23482 | Unknown | -0.305 | 3.6e-01 | 5.0e-01 |
| 337 | X - 23739 | Unknown | 0.403 | 2.4e-02 | 7.1e-02 |
| 338 | X - 23775 | Unknown | 0.213 | 2.2e-02 | 6.6e-02 |
| 339 | X - 24425 | Unknown | 0.250 | 1.4e-03 | 1.6e-02 |
| 340 | X - 24435 | Unknown | 0.671 | 2.2e-02 | 6.6e-02 |
| 341 | X - 24559 | Unknown | -0.128 | 7.8e-01 | 8.6e-01 |
| 342 | X - 24682 | Unknown | -0.153 | 9.7e-01 | 9.8e-01 |
| 343 | X - 24740 | Unknown | -0.095 | 7.1e-01 | 8.0e-01 |
| 344 | X - 24803 | Unknown | -0.132 | 9.3e-01 | 9.7e-01 |
| 345 | X - 24804 | Unknown | 0.094 | 3.8e-01 | 5.2e-01 |
| 346 | X - 24806 | Unknown | 0.043 | 5.1e-01 | 6.4e-01 |
| 347 | X - 24813 | Unknown | -0.157 | 4.1e-01 | 5.4e-01 |
| 348 | X - 13529 | Unknown | 0.124 | 3.1e-01 | 4.6e-01 |
|  | **P-values* >0.05 are indicated in bold |  |  |  |  |
