# Supplementary material for: Multi-omics analysis to decipher the molecular link between chronic exposure to pollution and human skin dysfunction
Source: Sci Rep. 2021 Sep 15;11:18302. doi: 10.1038/s41598-021-97572-1 (PMC8443591; doi:10.1038/s41598-021-97572-1)
Supplement: Supplementary file 4 — Supplementary Table 3. [file 41598_2021_97572_MOESM4_ESM.docx]

**Supplementary Table 3: Metabolites presenting correlation with bacterial and fungal diversity (shannon index).**

| **Pathway** | **Sub-Pathway** | **Metabolite** | **shannon_bacteria** | | **shannon_fungi** | |
| --- | --- | --- | --- | --- | --- | --- |
|  |  |  | **Pearson Correlation Coefficients** | ***P*-values*** | **Pearson Correlation Coefficients** | ***P*-values*** |
| Amino acid | Alanine and Aspartate Metabolism | alanine | 0.36 | **<0.0001** | -0.14 | 0.1057 |
| Amino acid | Lysine Metabolism | N6_acetyllysine | -0.48 | **<0.0001** | -0.10 | 0.2701 |
| Amino acid | Urea cycle; Arginine and Proline Metabolism | N_delta_acetylornithine | -0.49 | **<0.001** | -0.10 | 0.2718 |
| Amino acid | Histidine Metabolism | cis_urocanate | 0.32 | **0.0002** | -0.01 | 0.9519 |
| Amino acid | Tryptophan Metabolism | kynurenate | 0.29 | **0.0006** | -0.12 | 0.1534 |
| Amino acid | Histidine Metabolism | N_acetylhistidine | 0.28 | **0.0010** | 0.07 | 0.4366 |
| Amino acid | Glutathione Metabolism | _5_oxoproline | 0.28 | **0.0011** | -0.15 | 0.0897 |
| Amino acid | Glycine, Serine and Threonine Metabolism | N_acetylglycine | 0.28 | **0.0011** | 0.04 | 0.6151 |
| Amino acid | Leucine, Isoleucine and Valine Metabolism | methylsuccinate | 0.27 | **0.0017** | -0.10 | 0.2712 |
| Amino acid | Urea cycle; Arginine and Proline Metabolism | ornithine | 0.26 | **0.0028** | -0.03 | 0.7203 |
| Amino acid | Glycine, Serine and Threonine Metabolism | serine | 0.25 | **0.0032** | -0.12 | 0.1768 |
| Amino acid | Glycine, Serine and Threonine Metabolism | N_acetylserine | 0.23 | **0.0087** | -0.05 | 0.5838 |
| Amino acid | Glycine, Serine and Threonine Metabolism | glycine | 0.21 | **0.0175** | -0.08 | 0.3627 |
| Amino acid | Polyamine Metabolism | acisoga | 0.20 | **0.0192** | -0.01 | 0.8845 |
| Amino acid | Urea cycle; Arginine and Proline Metabolism | proline | 0.19 | **0.0259** | -0.07 | 0.4244 |
| Amino acid | Glycine, Serine and Threonine Metabolism | threonine | 0.19 | **0.0280** | -0.12 | 0.1654 |
| Amino acid | Leucine, Isoleucine and Valine Metabolism | isovalerylglycine | 0.18 | **0.0387** | 0.07 | 0.4382 |
| Amino acid | Leucine, Isoleucine and Valine Metabolism | isoleucine | 0.18 | **0.0391** | -0.09 | 0.3132 |
| Amino acid | Alanine and Aspartate Metabolism | asparagine | 0.17 | 0.0532 | -0.12 | 0.1734 |
| Amino acid | Urea cycle; Arginine and Proline Metabolism | citrulline | 0.16 | 0.0616 | -0.11 | 0.1943 |
| Amino acid | Leucine, Isoleucine and Valine Metabolism | valine | 0.16 | 0.0725 | -0.08 | 0.3357 |
| Amino acid | Histidine Metabolism | _4_imidazoleacetate | 0.15 | 0.0779 | -0.08 | 0.3418 |
| Amino acid | Alanine and Aspartate Metabolism | N_acetylaspartate | 0.15 | 0.0811 | -0.07 | 0.4402 |
| Amino acid | Creatine Metabolism | guanidinoacetate | 0.15 | 0.0887 | -0.10 | 0.2759 |
| Amino acid | Histidine Metabolism | histidine | 0.15 | 0.0953 | -0.18 | **0.0394** |
| Amino acid | Glutamate Metabolism | N_acetylglutamine | -0.14 | 0.1194 | 0.11 | 0.2080 |
| Amino acid | Leucine, Isoleucine and Valine Metabolism | N_acetylvaline | 0.12 | 0.1618 | -0.01 | 0.9183 |
| Amino acid | Histidine Metabolism | anserine | 0.12 | 0.1622 | 0.01 | 0.9014 |
| Amino acid | Alanine and Aspartate Metabolism | N_acetylalanine | 0.11 | 0.2044 | -0.08 | 0.3750 |
| Amino acid | Urea cycle; Arginine and Proline Metabolism | trans_4_hydroxyproline | -0.09 | 0.2997 | 0.29 | **0.0007** |
| Amino acid | Urea cycle; Arginine and Proline Metabolism | N_monomethylarginine | 0.09 | 0.3317 | -0.01 | 0.8709 |
| Amino acid | Leucine, Isoleucine and Valine Metabolism | leucine | 0.08 | 0.3370 | -0.08 | 0.3818 |
| Amino acid | Glycine, Serine and Threonine Metabolism | N_acetylthreonine | 0.04 | 0.6274 | -0.04 | 0.6235 |
| Amino acid | Urea cycle; Arginine and Proline Metabolism | _2_oxoarginine_ | 0.04 | 0.6428 | -0.12 | 0.1779 |
| Peptides | Dipeptide | leucylalanine | 0.14 | **0.0044** | 0.06 | 0.5120 |
| Peptides | Gamma-glutamyl Amino Acid | gamma_glutamylthreonine | 0.22 | **0.0126** | -0.18 | **0.0362** |
| Peptides | Gamma-glutamyl Amino Acid | gamma_glutamylalanine | 0.22 | **0.0130** | -0.15 | 0.0941 |
| Peptides | Gamma-glutamyl Amino Acid | gamma_glutamylglycine | 0.21 | **0.0163** | -0.18 | **0.0342** |
| Peptides | Gamma-glutamyl Amino Acid | gamma_glutamyltryptophan | 0.20 | **0.0201** | -0.19 | **0.0259** |
| Peptides | Gamma-glutamyl Amino Acid | gamma_glutamylserine | 0.20 | **0.0209** | -0.19 | **0.0297** |
| Peptides | Dipeptide | glycylisoleucine | 0.20 | **0.0222** | -0.09 | 0.2974 |
| Peptides | Gamma-glutamyl Amino Acid | gamma_glutamylvaline | 0.19 | **0.0252** | -0.15 | 0.0870 |
| Peptides | Gamma-glutamyl Amino Acid | gamma_glutamylglutamate | 0.18 | **0.0418** | -0.09 | 0.3194 |
| Peptides | Gamma-glutamyl Amino Acid | gamma_glutamylhistidine | 0.17 | **0.0480** | -0.20 | **0.0218** |
| Peptides | Gamma-glutamyl Amino Acid | gamma_glutamyltyrosine | 0.15 | 0.0897 | -0.16 | 0.0717 |
| Peptides | Gamma-glutamyl Amino Acid | gamma_glutamylisoleucine_ | 0.14 | 0.1102 | -0.16 | 0.0681 |
| Peptides | Gamma-glutamyl Amino Acid | gamma_glutamyl_alpha_lysine | 0.12 | 0.1624 | -0.10 | 0.2649 |
| Peptides | Gamma-glutamyl Amino Acid | gamma_glutamylleucine | 0.12 | 0.1882 | -0.15 | 0.0793 |
| Peptides | Gamma-glutamyl Amino Acid | gamma_glutamylphenylalanine | 0.11 | 0.2187 | -0.15 | 0.0813 |
| Peptides | Gamma-glutamyl Amino Acid | gamma_glutamylglutamine | 0.09 | 0.3266 | -0.10 | 0.2434 |
| Lipid | Fatty Acid, Dicarboxylate | maleate | 0.37 | **<0.0001** | -0.06 | 0.4870 |
| Lipid | Fatty Acid Synthesis | malonate | 0.31 | **0.0003** | 0.06 | 0.5114 |
| Lipid | Mevalonate Metabolism | _3_hydroxy_3_methylglutarate | 0.29 | **0.0007** | 0.03 | 0.7578 |
| Lipid | Fatty Acid, Dicarboxylate | _3methylglutarate_2_methylglutarate | 0.29 | **0.0009** | -0.01 | 0.9334 |
| Lipid | Fatty Acid, Amide | oleamide | 0.27 | **0.0019** | -0.15 | 0.0958 |
| Lipid | Fatty Acid, Dicarboxylate | undecanedioate_C11_DC | 0.25 | **0.0046** | 0.14 | 0.1023 |
| Lipid | Long Chain Fatty Acid | _2_hydroxydecanoate | 0.19 | **0.0337** | -0.06 | 0.5024 |
| Lipid | Fatty Acid, Dicarboxylate | azelate_C9_DC | 0.18 | **0.0380** | 0.14 | 0.1214 |
| Lipid | Fatty Acid, Dicarboxylate | _2_hydroxyadipate | 0.16 | 0.0738 | -0.01 | 0.9191 |
| Lipid | Endocannabinoid | linoleoyl_ethanolamide | 0.14 | 0.1170 | -0.07 | 0.4254 |
| Lipid | Diacylglycerol | linoleoyl_linoleoyl_glycerol_18 | -0.10 | 0.2720 | 0.04 | 0.6255 |
| Lipid | Fatty Acid, Dicarboxylate | dodecanedioate_C12_DC | 0.08 | 0.3698 | -0.01 | 0.8926 |
| Lipid | Fatty Acid, Monohydroxy | _2_hydroxystearate | 0.02 | 0.8122 | -0.04 | 0.6871 |
| Lipid | Polyunsaturated Fatty Acid (n3 and n6) | linoleate_18_2n6 | 0.02 | 0.8530 | -0.27 | **0.0015** |
| Carbohydrate | Glycolysis, Gluconeogenesis, and Pyruvate Metabolism | lactate | 0.25 | **0.0044** | -0.02 | 0.8270 |
| Energy | TCA Cycle | fumarate | 0.26 | **0.0030** | -0.02 | 0.8421 |
| Energy | TCA Cycle | citraconate_glutaconate | 0.28 | **0.0014** | -0.02 | 0.8375 |
| Cofactors and Vitamins | Vitamin B6 Metabolism | pyridoxate | 0.06 | 0.4835 | -0.06 | 0.5106 |
| Xenobiotics | Food Component/Plant | piperine | -0.17 | **0.0473** | 0.07 | 0.4144 |
| Xenobiotics | Chemical | thioproline | -0.29 | **0.0009** | 0.05 | 0.5692 |
| Xenobiotics | Tobacco Metabolite | nicotine | -0.10 | 0.2730 | 0.24 | **0.0066** |
| Unknown | Unknown | X_13737 | 0.25 | **0.0034** | -0.27 | **0.0018** |
| Unknown | Unknown | X_22102 | 0.16 | 0.0667 | -0.06 | 0.5110 |
| Unknown | Unknown | X_23739 | 0.14 | 0.1034 | -0.21 | **0.0139** |
| Unknown | Unknown | X_13529 | 0.14 | 0.1198 | -0.07 | 0.4270 |
| Unknown | Unknown | X_13504 | 0.14 | 0.1213 | -0.12 | 0.1773 |
| Unknown | Unknown | X_14904 | 0.10 | 0.2611 | 0.12 | 0.1851 |
| Unknown | Unknown | X_16267 | 0.09 | 0.3042 | -0.07 | 0.4249 |
| Unknown | Unknown | X_23196 | 0.06 | 0.4613 | 0.13 | 0.1468 |
| **P-values* >0.05 are indicated in bold | |  |  |  |  |  |
