# Supplementary material for: Multi-omics analysis to decipher the molecular link between chronic exposure to pollution and human skin dysfunction
Source: Sci Rep. 2021 Sep 15;11:18302. doi: 10.1038/s41598-021-97572-1 (PMC8443591; doi:10.1038/s41598-021-97572-1)
Supplement: Supplementary file 5 — Supplementary Table 4. [file 41598_2021_97572_MOESM5_ESM.rtf]

Supplementary Table 4: Lifestyle Questionnaire and Demographic Details

Life Style Questionnaire	INTENSITY	DALIAN (count)	DALIAN (%)	BAODING (count)	BAODING (%)	P-value	
How often do you wear a mask to protect your face from pollution?	No	37	55.224	22	32.836	0.0099	
	On polluted days peaks	10	14.925	15	22.388		
	Sometimes	18	26.866	22	32.836		
	Every time you are outside	2	2.985	8	11.940		
How often do you wear a hat to protect your hair from pollution?	No	34	50.746	36	53.731	0.7847	
	High UV exposure	4	5.970	3	4.478		
	On polluted days peaks	2	2.985	4	5.970		
	Sometimes	26	38.806	21	31.343		
	Every time you are outside	1	1.493	3	4.478		
Daily time spent outdoor during the day (hobbies, transportation, work ...)	Less than 2 hours a day	25	37.313	29	43.284	0.7466	
	Between 2 and 4 hours a da	39	58.209	32	47.761		
	More than 4 hours a day	3	4.478	6	8.955		
Time spent outdoor before 18 years of age (school, transportation, outside games	Less than 2 hours a day	9	13.433	16	23.881	0.0923	
	Between 2 and 4 hours a da	33	49.254	33	49.254		
	More than 4 hours a day	25	37.313	18	26.866		
Working environment	Mainly indoor (outside les	67	100.000	67	100.000	1	
Sunbathing frequency	No sunbathing	30	44.776	65	97.015	<.0001	
	Few sunbathing	30	44.776	2	2.985		
	Sometimes sunbathing	7	10.448	0	0		
Red meat	Less or Never red meat	30	44.776	28	41.791	0.4281	
	1-3 times red meat /week	36	53.731	33	49.254		
	4-7 times red meat /week	1	1.493	6	8.955		
Fish/Seafood	Less or Never fish/Seafood	9	13.433	36	53.731	<.0001	
	1-3 times fish/Seafood /we	50	74.627	30	44.776		
	4-7 times fish/Seafood /we	8	11.940	1	1.493		
You prefer to eat: Spicy	Few or Not spicy	33	49.254	33	49.254	0.8041	
	Lightly spicy	25	37.313	28	41.791		
	Very spicy	9	13.433	6	8.955		
You prefer to eat: Salty	Few or Not salty	31	46.269	29	43.284	0.9545	
	Lightly salty	29	43.284	35	52.239		
	Very salty	7	10.448	3	4.478		
You prefer to eat: Sweet	Few or Not Sweet	26	38.806	26	38.806	0.6182	
	Lightly Sweet	30	44.776	35	52.239		
	Very Sweet	11	16.418	6	8.955		
What kind of water do you drink: Tap Water	No tap water	6	8.955	27	40.299	<.0001	
	Tap water	61	91.045	40	59.701		
What kind of water do you drink: Purified Water	No purified water	21	31.343	24	35.821	0.5832	
	Purified water	46	68.657	43	64.179		
How often do you cook?	Rarely cook	40	59.701	22	32.836	0.0009	
	Often cook	19	28.358	24	35.821		
	Everyday cook	8	11.940	21	31.343		
you have a hood in your kitchen	No hood in kitchen	0	0	4	5.970	0.0423	
	Hood in kitcken	67	100.000	63	94.030		
What is your main kind of cooking?	Boiled	18	26.866	62	92.537	<.0001	
	Fried	49	73.134	5	7.463		
How often do you heat your house?	From 1 month to 3 months	0	0	1	1.493	<.0001	
	From 3 months to 6 months	67	100.000	3	4.478		
	6 months or more	0	0	63	94.030		
Do you have a switch on heating system in your bedroom?	No heating system in your	59	88.060	30	44.776	<.0001	
	Heating system in your bed	8	11.940	37	55.224		
How do you heat your house? (Several answers allowed) - Coal	No coal	0	0	27	40.299	<.0001	
	Coal	67	100.000	40	59.701		
How do you heat your house? (Several answers allowed) - Fuel oil	No fuel oil	67	100.000	67	100.000	1	
How do you heat your house? (Several answers allowed) - Gas	No gas	67	100.000	46	68.657	<.0001	
	Gas	0	0	21	31.343		
How do you heat your house? (Several answers allowed) - Electric	No electric	57	85.075	59	88.060	0.6124	
	Electric	10	14.925	8	11.940		
Age	25-30	15	22.388	17	25.373	0.7672	
	30-35	15	22.388	14	20.896		
	35-40	18	26.866	18	26.866		
	40-45	19	28.358	18	26.866		
Education	Technicals	8	11.940	0	0	<.0001	
	High School	7	10.448	30	44.776		
	College	18	26.866	35	52.239		
	Bachelor	30	44.776	2	2.985		
	Master	4	5.970	0	0		
BMI	<18.5	4	5.970	8	11.940	0.6578	
	18.5-25	51	76.119	41	61.194		
	25-30	11	16.418	16	23.881		
	>30	1	1.493	2	2.985		
